# A Dynamic View of Domain-Motif Interactions

**DOI:** 10.1371/journal.pcbi.1002341

**Published:** 2012-01-12

**Authors:** Eyal Akiva, Gilgi Friedlander, Zohar Itzhaki, Hanah Margalit

**Affiliations:** Department of Microbiology and Molecular Genetics, IMRIC, Faculty of Medicine, The Hebrew University of Jerusalem, Jerusalem, Israel; University of Zurich and Swiss Institute of Bioinformatics, Switzerland

## Abstract

Many protein-protein interactions are mediated by domain-motif interaction, where a domain in one protein binds a short linear motif in its interacting partner. Such interactions are often involved in key cellular processes, necessitating their tight regulation. A common strategy of the cell to control protein function and interaction is by post-translational modifications of specific residues, especially phosphorylation. Indeed, there are motifs, such as SH2-binding motifs, in which motif phosphorylation is required for the domain-motif interaction. On the contrary, there are other examples where motif phosphorylation prevents the domain-motif interaction. Here we present a large-scale integrative analysis of experimental human data of domain-motif interactions and phosphorylation events, demonstrating an intriguing coupling between the two. We report such coupling for SH3, PDZ, SH2 and WW domains, where residue phosphorylation within or next to the motif is implied to be associated with switching on or off domain binding. For domains that require motif phosphorylation for binding, such as SH2 domains, we found coupled phosphorylation events other than the ones required for domain binding. Furthermore, we show that phosphorylation might function as a double switch, concurrently enabling interaction of the motif with one domain and disabling interaction with another domain. Evolutionary analysis shows that co-evolution of the motif and the proximal residues capable of phosphorylation predominates over other evolutionary scenarios, in which the motif appeared before the potentially phosphorylated residue, or vice versa. Our findings provide strengthening evidence for coupled interaction-regulation units, defined by a domain-binding motif and a phosphorylated residue.

## Author Summary

Domain-motif interactions are instrumental for many central cellular processes, and are therefore tightly regulated. Phosphorylation events are known modulators of protein-protein interactions in general, including domain-motif interactions. Here, we addressed the association of phosphorylation and domain-motif interaction taking a motif-centred view. We integrated human domain-motif interaction and phosphorylation data for four representative domains (SH2, WW, SH3 and PDZ), and showed that the adjacency between phosphorylation and domain-motif interactions is extensive, suggesting interesting functional links between them that extend the classical and widely studied phospho-regulation of SH2 or WW domain-motif interactions. Furthermore, we show that such interaction-regulation units may function as double switches, concurrently enabling interaction of the motif with one domain and disabling interaction with another domain. These latter interaction-regulation units are more conserved in evolution than the individual units comprising them. Assuming that the four analyzed domain-motif interaction types are reliable representatives of such interactions, our results support the existence of units comprising motifs and associated phosphorylation sites, in which the regulation of domain-motif interaction is inherent.

## Introduction

The *modus operandi* of cellular machinery is fundamentally dependent on the intricate network of physical associations between proteins. Hence, deciphering the basic details of this network, the interacting protein pairs and the protein elements mediating the interaction, is a major challenge. In the last decade it became widely accepted that protein domains play a key role in mediating protein-protein interactions. A prominent type of domain-mediated protein-protein interaction is domain-motif interaction, commonly achieved by a domain in one protein and a short linear motif in the interacting partner [1]. These interactions, frequently of transient nature, play a major role in cellular processes, such as signal transduction and protein targeting to cellular compartments [2]. Distinct domains are known to interact with specific motifs, where both the motif and the domain are typified by their sequences (*e.g.* interactions between SH3 domains and proline-rich motifs [3]). Motifs are short protein regions (typically 3–10 residues) that frequently match a specific sequence pattern [4]. Usually, this pattern confines two or three positions that are essential for the interaction with the corresponding domain, while other positions are less restricted. This loosely confined sequence pattern leads to intricate interaction relationships between domains and motifs. For example, several domains from the same family may bind a single motif in one protein. Moreover, same-family domains may bind different variations of the same motif. For instance, PDZ domains may bind different motifs at the C-termini of their interacting partners, such as class I (x[S/T]xΨ-COOH), class II (xΨxΨ-COOH) or class III (x[E/D]xΨ-COOH) motifs, where x is any residue and Ψ is a hydrophobic residue [5]. All these characteristics of domain-motif interactions may hint at a network of promiscuous associations. Nevertheless, domain-motif interactions display specificity that stems from various factors. For instance, residues other than the ones restricted by the sequence pattern may set the interaction specificity of motifs of the same type. In addition, residues in the binding cleft of the domain contribute to specificity. Importantly, the sequence context of the motif also plays a role in interaction specificity [6], [7], [8]. Hence, the motif's sequence pattern serves as a scaffold for the interaction, while contextual spatial and temporal information contributes to interaction specificity [4].

The comprehensive involvement of domain-motif interactions in key cellular processes necessitates tight regulation. Protein phosphorylation is well-accepted as a generic regulator of protein-protein interactions, including domain-motif interactions [7], [9]. A protein phosphorylation event may affect the protein's activity, stability, localization or interaction potential by inducing a conformational change or by forming/preventing a binding site for other molecules [10]. Phosphorylation may affect domain-motif interactions in two major ways: (a) It turns ‘on’ interactions for domains that are known to interact with motifs only when they are phosphorylated (*e.g.* SH2 and class IV WW domains [2], [11]), and (b) It may serve as an ‘off’ switch for domains that bind un-phosphorylated motifs (*e.g.* SH3 and PDZ domains). The phospho-regulation of the former has been studied extensively while the phospho-regulation of the latter has been noted in sporadic cases. For instance, the interaction between NCK and PAK1, which is mediated by SH3-motif interaction, is prevented by phosphorylation of a residue just near the motif [12].

Here we study this regulatory mechanism, focusing especially on motifs in which phosphorylation is not required for domain binding, but rather might play a preventive role. The results of our large-scale integrative study point to the existence of coupled interaction-regulation units, where phosphorylation within or near the motif is suggested to play a role as an ‘on’/‘off’ switch of domain-motif interactions.

## Results

### Evidence for coupling between motifs and phosphorylation events

We chose human as the organism for our study, due to the wealth of phosphorylation and domain-motif interaction experimental data. First, we generated a comprehensive database of protein phosphorylation events derived from nine data sources (Table 1 and Methods), where we recorded experimentally-determined phosphorylated residues. Next, we unified seven resources of experimentally-verified domain-motif interactions, including motifs that bind any of the domains SH2, WW, SH3 and PDZ. Each of these two databases was further categorized according to evidence reliability (Table 1 and Methods). All reported phosphorylation events and all motifs derived from the different databases were mapped onto the human proteome derived from the Uniprot database (see Methods). We refer to all of the documented short sequence stretches that bind domains as motifs, even though the sequence pattern acknowledged as representing the domain-binding motif could be identified in only 81% of them (see Methods).

**Table 1:** Phosphorylation and domain-motif interaction databases.

Phosphorylation database			
	LTP	HTP	Total
# phosphorylated residues	8,472	50,544	59,016
# phosphorylated proteins	2,555	8,570	9,322

Domain-motif interaction database*			
Domain	LTP	HTP	Total
SH2	552 (330)	821 (152)	1,373
WW	112 (62)	107 (58)	219
PDZ	114 (89)	7 (6)	121
SH3	89 (70)	881 (308)	970

Abbreviations: LTP (low throughput experimental evidence), HTP (high throughput experimental evidence).

*Counts of domain-motif interactions. Since a single motif may bind multiple domains of the same family, the non-redundant counts of domain-binding motifs were added (in parentheses).

**Figure 1:**
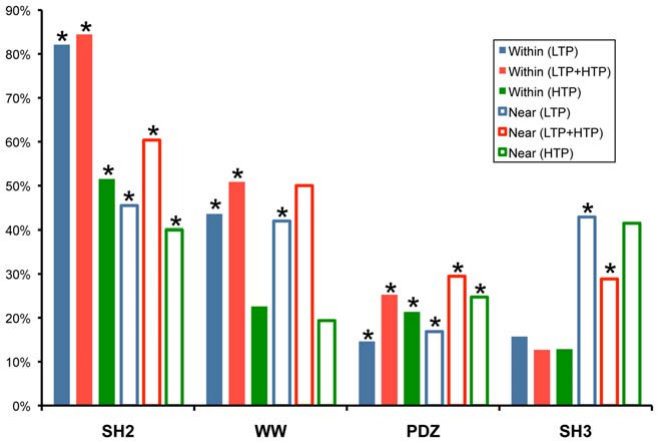
Coupling between phosphorylation events and domain-binding motifs. For each domain family (SH2, WW, PDZ and SH3), the bars denote the percent of motifs found to be phosphorylated either within or near them. Solid-colored and empty rectangular bars represent intra-motif phosphorylation and near-motif phosphorylation, respectively. All motifs are derived from the high reliability dataset, while phosphorylation events are derived from three data sets: LTP (low throughput evidence only), HTP (phosphorylation events based on evidence from high-throughput resources), and LTP+HTP (any type of evidence). Asterisks represent statistically-significant results (Methods, Table 1 and Table S1).

For all four domain-motif interaction types we integrated the data of bound motifs with the data of phosphorylated residues, searching for phosphorylated residues either within or near any given motif. We defined the vicinity of the motif as its N-terminal and C-terminal 20 flanking residues. This was based on the length of the disordered context of motifs [13]. We verified that our motifs are indeed situated within a disordered sequence context that spans even more than the 20 residues in each side of the motif (Figure S1). A recent work that studied co-evolution between motifs and their context [6] used the same length. To assess the statistical significance of the motif-phosphorylation coupling we compared the number of such events in our data to that found in randomized datasets. As demonstrated in Figure 1, there is a statistically significant association between motifs and phosphorylation events, evident for the vast majority of motif types and throughout the various levels of data reliability (see Methods and Table S1). This regards both phosphorylation events within the motifs and near the motifs. Of note, the average distance between motifs and nearby phosphorylation events is similar in the actual and random datasets, but the abundance of coupled events is higher in the actual data. To verify that our data are not skewed because of database tendency to include motifs and their functionally associated phosphorylation sites (*e.g.* a database curator will naturally find one reference with a documented motif that binds an SH2 domain, along with the relevant phospho-tyrosine), we repeated our randomization test using only high-throughput phosphorylation data and low-throughput motif data. In the majority of the cases, the association between motifs and phosphorylation sites remained statistically significant (Figure 1 and Table S1). These results further support the association between motifs and phosphorylation sites.

Since SH2 domains bind motifs with phosphorylated residues [2], we expect many of these motifs to include a documented phosphorylated residue within them. Indeed, 83% of the SH2-binding motifs included a documented phosphorylated tyrosine residue. We also observed a statistically significant and high overlap (52%) between SH2-motifs derived from low-throughput experiments and phosphorylation data from only high-throughput experiments (Figure 1 and Table S1). These results support the reliability of our motif and phosphorylation data integration, since we took these two different sources of information independently and got a high percent of phosphorylation sites that are required for SH2 interaction. Still, for 17% of the SH2-binding motifs we did not identify a phosphorylated tyrosine as expected. Examination of these cases revealed that in most of them the original evidence for SH2 domain binding was based on tyrosine to phenylalanine mutation that abolished the domain-motif interaction. This kind of experiment does not supply a direct evidence for phospho-tyrosines and therefore these phosphorylation events were missing from our low-throughput data. The binding of class IV WW domains to their respective motifs is also known to require motif phosphorylation. This implies that over-representation of intra-WW motif phosphorylation is expected. Our data integration for the WW-binding motifs revealed that 46% of these motifs were also found to include a phosphorylated serine or threonine. While the specific WW-domain class is not annotated in our domain-motif interaction database, the fact that 61/120 motifs were phosphorylated and 55/61 out of these motifs obey a previously characterized class IV WW motif (based on ELM [14] and NetPhorest [4] prediction), further supports the quality of our data integration.

Interestingly, 150 out of the highly reliable 330 SH2-binding motifs (*i.e.* based on low-throughput methodologies, see Methods) had highly reliable phosphorylated residues in their vicinity (≤20 residues), pointing at potential functional implications that require further investigation. In 60% of these cases, the nearby phosphorylated residue was tyrosine (this percent greatly deviates from the overall percent of phospho-tyrosines in our data, which is only 15%). The fraction of tyrosines among all residues in the flanking regions of SH2-binding motifs (3.4%) is statistically significantly higher than their fraction in the human proteome (2.7%, p = 3.85e-9 by Fisher exact test). Notably, 57.4% of the tyrosines near SH2-binding motifs are phosphorylated, whereas only 2.9% of all tyrosines in the human proteome are phosphorylated. It might be that this phosphorylation is auxiliary to the tyrosine phosphorylation of the binding motif. However, the higher frequency of tyrosines in the vicinity of SH2-binding motifs may suggest that their role is to attract the tyrosine kinase to this region. By this interpretation, the nearby phosphorylation of tyrosine enhances the phosphorylation needed for the motif to bind SH2.

Our analysis of SH3- and PDZ-bound motifs, most of which are known to bind the corresponding domains when they are not phosphorylated, identified a statistically significant coupling between these motifs and phosphorylation events (Figure 1). Conceivably, for SH3- and PDZ-binding motifs phosphorylation may prevent domain binding. To further support this conjecture we searched the literature for documented cases of functional relationship between motifs and phosphorylation events. Indeed we found several examples for phosphorylation within the motif or in its vicinity that prevents interaction of PDZ-, SH3- and WW-binding motifs to their respective domains. For example, the interaction between WW class I domains and motifs that match the PPxY sequence pattern may be prevented by tyrosine phosphorylation [15]. Likewise, tyrosine phosphorylation near a motif in the ErbB2 protein significantly reduced the motif's binding affinity to the PDZ domain in ERBIN [16]. Other examples of the effect of intra- or near-motif phosphorylation are detailed in Table 2. All the identified interaction-regulation units are detailed in Dataset S1 and available on http://margalit.huji.ac.il/PLoS_CB_supplemental_datasets.xls.gz.

**Table 2:** Experimental evidence of phosphorylation-mediated modulation of domain-motif interactions.

Domain type	Domain-containing protein	Motif-containing protein	Phosphorylated residue	Intra/near motif	Reference
PDZ	PSD-95	Kir2.3	S440	Intra	[78]
	PSD-95	Beta-1-adrenergic receptors	Various	Intra	[79]
	PSD-95	Kir5.1	S417	Intra	[80]
	PSD-95	Stargazin	T321	Intra	[26]
	EBP-50	ß2-adrenergic receptor	S411	Intra	[81]
	Syntenin-1	syndecan-1	T309	Intra	[82]
	Syntenin-1	syndecan-4	S183	Intra	[27]
	AF6, ERBIN, SNA1	Various	Various	Intra	[83]
	ERBIN	ErbB2	T1248	Near* ^(3)^	[16]
	Grasp65	Grasp65	S189	Near^(20)^	[84]
SH3	Syndapin-1	Dynamin-1	S774	Intra	[85]
	Endophilin-1	Dynamin-1	S778	Intra	[86]
	Nck	Pak1	S21	Near^(5)^	[12]
	Fyn	Tau	T231	Near^(7)^	[76]
	Fyn	Tau	S210	Near^(3)^	[76]
WW	Utrophin	ß-dystroglycan	Y892	Intra	[15]
	Various	Smad2, Smad3	S208, S204	Near^(17,21)^	[87]

*Numbers in parentheses indicate the distance between the motif and the proximal phosphorylation site/s.

Our findings encouraged us to search for additional evidence for coupled motifs and phosphorylation sites in organisms other than human. To this end, we needed reliable data of domain-motif interactions in other organisms. While such data are very scarce, we succeeded to find SH3-binding data from a large-scale experiment in *Saccharomyces cerevisiae* [17]. Integration of these data with phosphorylation data (Methods, Dataset S2) revealed a statistically significant association between SH3 motifs and phosphorylation sites (61 cases of phosphorylation sites either within or near motifs, p<0.0027 for near-motif phosphorylation). These results further extend the conclusions based on the human data. In summary, the positional association between motifs and phosphorylation events, backed by their potential functional coupling, allows us to suggest a new interaction-regulation unit, encompassing the motif and the phosphorylated residue.

### Phosphorylation as a specificity switch

The coupling between phosphorylation and motifs highlights the well-established switch-like function of phosphorylation, turning on or off the interaction, depending on the domain-motif interaction type. Intriguingly, phosphorylation of a motif may also serve as a double switch, concurrently switching ‘on’ the interaction with one domain and switching ‘off’ the interaction with a different domain. We tried to find evidence for such double switches for motifs that bind domains that belong to different families (section I below) or domains of the same family (section II below).

#### I. Specificity switch for motifs that bind two domains of different families

**Figure 2:**
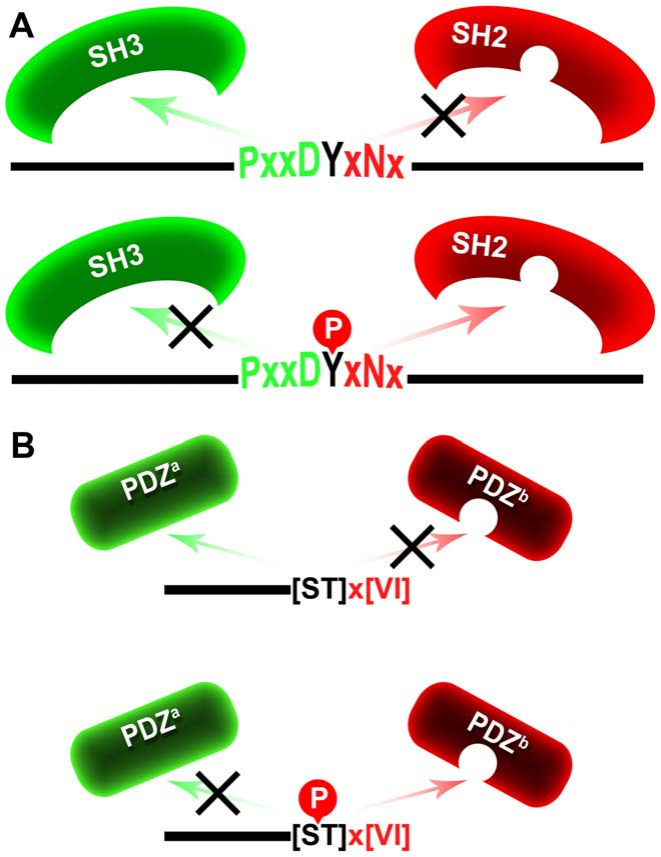
Phosphorylation events as double switches. (**A**) A protein (black horizontal line) includes a segment that matches two sequence patterns: the first is typical for SH3 domain binding (green), and the second typifies SH2 domain binding (red). The non-phosphorylated form binds SH3 and not SH2 (upper), while phosphorylation inverts the binding preferences (lower). (**B**) Specificity switches within the PDZ domain family. A protein (black horizontal line) includes a segment that may bind distinct PDZ domains (upper). The non-phosphorylated form binds PDZ^a^ and not PDZ^b^, while phosphorylation inverts these binding preferences (lower).

**Figure 3:**
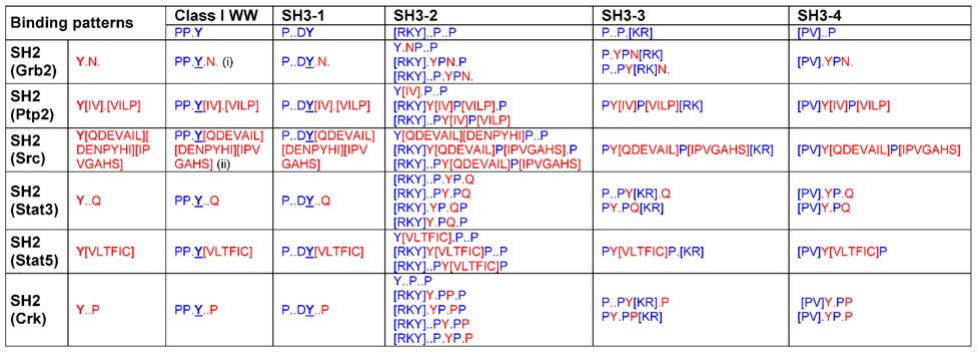
Dual sequence patterns used for the identification of potential double switches in human proteins. Column titles include sequence patterns for motifs that bind SH3 or class I WW domains (in red), and row titles include sequence patterns for motifs that bind different types of SH2 domains, upon motif phosphorylation (in blue). Each table cell includes a merged sequence pattern that hints at a dual binding potential of the motif to both SH2 and SH3 (or WW) domains. The columns under class I WW and SH3-1 titles represent the strict analysis scheme. Sequence patterns were extracted from the ELM database [14]. (i) An example for a dual motif. The PP.Y.N. sequence pattern is composed of the SH2^Grb2^ Y.N. and the class I WW PP.Y patterns. (ii) Note that this sequence pattern encompasses seven positions.

Consider the following scenario: a protein segment includes two merged motifs: an SH2-binding motif (*e.g.* YxNx pattern) and an SH3- binding motif (*e.g.* PxxDY pattern) [14]. Combination of these two motifs yields a dual motif with the PxxDYxNx sequence pattern, which is capable of binding two different domains (SH2 and SH3) in a mutually exclusive manner. Tyrosine phosphorylation enables SH2 binding while preventing SH3 binding, and tyrosine de-phosphorylation enables SH3 binding while preventing SH2 binding. This phosphorylation may be regarded as a “double switch” (Figure 2A). Indeed, mining our data and the relevant literature yielded experimentally verified cases of SH2-SH3 and SH2-class I WW double switches (Table 3). We next turned to identify novel potential double switches in the human proteome. We defined all possible dual patterns as described above (SH2 with SH3 and SH2 with class I WW, see Figure 3), and searched for hits in all human protein sequences that include a phosphorylated residue according to our data. Our analysis was split into a strict scheme and a less strict scheme. In the strict analysis, we used the combination of SH2 patterns and SH3 patterns that include a tyrosine residue (meaning this amino acid is vital for the interaction with SH3 domain, see Figure 3, column 4). The same residue is also the one known to be phosphorylated in the SH2 motif pattern. In this way, we increase the confidence that the phosphorylation inhibits the interaction with the SH3 domain. Similarly, binding sequence patterns of SH2 and class I WW domains (the only WW pattern that has a tyrosine, Figure 3, column 3) were combined. In the second analysis scheme, more permissive definitions of the dual motifs were applied. This analysis included any overlapping sequence patterns between SH2 and SH3 or SH2 and WW sequence patterns (Figure 3). Importantly, all along we regarded only the cases in which we found a phosphorylated residue in a relevant position based on our data. This analysis revealed 57 and 187 putative double switches by the strict and less-strict analysis, respectively (Table S2). By both analyses, about 20% of the putative double switches involved highly-reliable phosphorylation events (*i.e.* based on low-throughput methods). These results strengthen the conjecture that motif phosphorylation may function as a double switch for binding domains from different families.

**Table 3:** Mutually exclusive binding of domain pairs to the same protein segment.

		Protein and domain names		
Name of the protein with the dual motifs	Position and sequence	Binding upon phosphorylation	Binding upon de-phosphorylation	References
CD3ε	162 PNPD**Y**EP**I** 169	Zap70 (SH2)	Eps8L1 (SH3)	[88]
ARMS/Kidins220	1089 PPRPPSG**Y**SQ**P** 1099	CrkL (SH2)	CrkL (SH3)	[89]
Beta-Dystroglycan	887 PPP**Y**VP**P** 893	c-Src (SH2)	Dystrophin (Class I WW)	[90]
Growth hormone receptor	534 **Y**FCEADAKKCIPVAP 548	STAT5 (SH2)	Nck1 (SH3)	[91]–[93]
Cbl	540 RDLPPPPPPDRP**Y**SVG 555	Fyn (SH2)	Src (SH3)	[94], [95]

Summary of literature-documented double switches. The second column includes protein sequences, where residues vital for SH2 binding and residues vital for SH3/class I WW binding are in bold and underlined, respectively. Rows (1–3) describe experimentally-verified double switches. Rows (4–5) include examples for which there is evidence for the motif binding to each domain, but not for a direct switch. Note that Y534 in growth hormone receptor is phosphorylated according to a high-throughput experiment. Also note that evidence for Fyn-Cbl interaction exists for the Cbl (552–614) fragment (spanning 62 residues), where Y552 is the only tyrosine, suggesting that this tyrosine is bound by the SH2 domain in Fyn.

To substantiate the association between the identified double switches and pairs of proteins, one carrying SH2 domain and the other carrying SH3 or class I WW domain, we turned to analyze large-scale data of protein-protein interaction in human. These data were derived from MINT, IntAct and DIP databases [18], [19], [20]. We identified in the network proteins that interact with pairs of proteins, such that one carries SH2 domain and the other carries SH3 or class I WW domain, and found that their overlap with proteins carrying dual motifs (strict scheme) was statistically significant (p-values of 1.9e-06 and 2.5e-08 for the SH2/SH3 and SH2/WW class I, respectively). Thus, proteins containing dual SH2/SH3 motifs are found to interact with protein pairs carrying SH2 domain in one protein and SH3 in the other more than expected at random (and the same holds for SH2/WW). Table S3 lists all these identified cases. Of note, the analysis of the whole network re-discovered three out of the five literature-documented cases of double-switches (Table 3, rows 1, 2 and 5).

47 of the double switches include highly reliable phosphorylated residues. Evolutionary analysis of these 47 double switches revealed that the sequence patterns of the two motifs co-appeared in the same phylogenetic branch in 55% of the cases. The other cases suggest an interesting stepwise appearance of switched binding: there are 14 cases in which an SH2 sequence pattern hit preceded SH3/class I WW pattern hits, and seven cases in which the order of appearance was opposite.

#### II. Specificity switch for motifs that bind domains of the same family

There are reports on proteins carrying the PDZ domain that require motif phosphorylation for binding [21], [22], [23]. This hints at double switches or specificity switches, within the same domain family (Figure 2B). To further explore this postulate we used a recently published proteome-wide interaction map for PDZ-motif interactions in mouse [24], [25]. For PDZ domains and C-terminal peptides this study recorded experimentally determined affinity values when an interaction occurred, and reported also when an interaction did not occur. Since data of phosphorylated and non-phosphorylated peptide interactions were not available in this study, we used the widely accepted assumption that substitution of Asp/Glu for Ser/Thr/Tyr residues may mimic the phosphorylated state of the latter, shown to be valid also for PDZ binding motifs [22], [26], [27], [28]. We chose peptide couples that display high sequence similarity, except for a single position in which a Ser/Thr/Tyr aligns with an Asp/Glu residue (‘pseudo-phosphorylated’ peptides). We found 81 cases of double switches, where two PDZ-containing proteins show inverse affinities to the non-phosphorylated and pseudo-phosphorylated peptides (see Methods and Table S4). This result is highly statistically significant (p≤0.0031, see Methods and Table S5). Next, we tried to find cases in which the Ser/Thr/Tyr residues of the PDZ-binding motif are documented as phosphorylated residues in mouse. Intriguingly, we found such evidence for positions 1005 and 1006 of Glutamate receptor delta-2 subunit (where phosphorylation is shown also to prevent PDZ binding) and position 913 of Atp2b1 [29], [30]. We also tried to map the predicted PDZ switches to human PDZ-motif interactions. We found that the putative switching residue is phosphorylated in several orthologous human proteins (orthologs were derived from the Inparanoid database [31]). The human 5-hydroxytryptamine receptor 2C is an ortholog of the htr2c mouse protein, where Ser 457 is documented as a phosphorylated residue. This phosphorylation is also shown to prevent PDZ domain binding [28]. Supporting evidence was also found for Ser 832 of Semaphorin-4C, the human ortholog of sema4c [32], and for Thr 321 of CACNG2, the human ortholog of stargazin [26]. The supporting evidence based on mouse and human data hint at 10 PDZ motifs that may serve as candidates for future studies. Altogether, our results support the notion that motif phosphorylation plays a role as a double switch also for different proteins carrying the same domain type, with implications to both human and mouse PDZ-motif interactions.

### Evolution of motif-phosphorylation coupling

We followed the evolutionary history of the interaction-regulation unit components by examining the human motif sequences and phosphorylation sites in orthologous proteins present in 15 eukaryotic organisms. We focused on human proteins that include units with motifs that match a previously characterized sequence pattern (see Methods). Each such protein was aligned with its orthologs (if they could be identified) in other organisms: *Pan troglodytes*, *Mus musculus*, *Rattus norvegicus*, *Bos taurus*, *Gallus gallus*, *Danio rerio*, *Xenopus tropicalis*, *Ciona intestinalis*, *Drosophila melanogaster*, *Anopheles gambiae*, *Caenorhabditis elegans*, *S. cerevisiae*, *Dictyostelium discoideum*, *Arabidopsis thaliana* and *Plasmodium falciparum*. Due to the scarcity of documented experimentally-based binding motifs in organisms other than human, we checked if the positions in the ortholog that are aligned with the human motif comply with a relevant sequence pattern. We also examined the conservation of the phosphorylation site in the ortholog by checking if it maintained in the corresponding positions the same residue as in human, or kept the phosphorylation potential (Ser/Thr/Tyr in the corresponding position).

**Figure 4:**
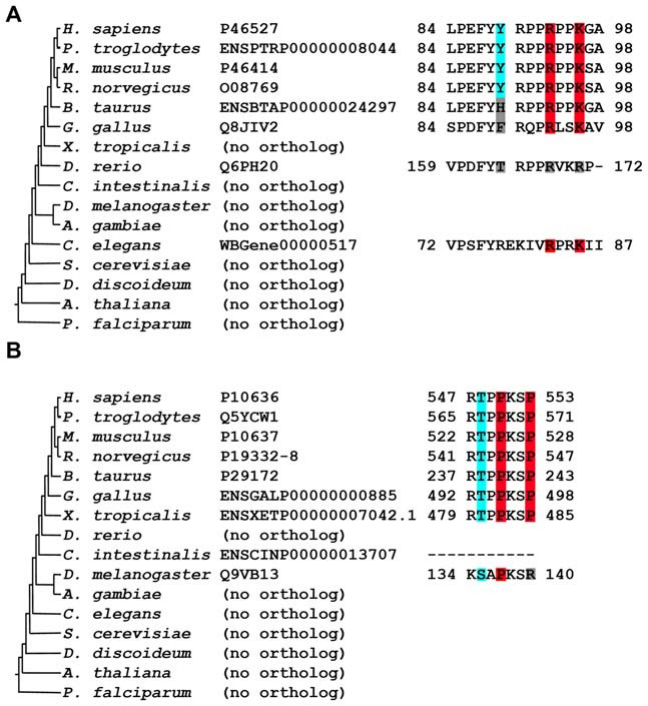
Step-wise appearance of motifs and potential phosphorylation sites. (**A**) The motif is older than the potential phosphorylation site. The human CDK inhibitor 1B (top line) includes an SH3-binding motif (RxxK, highlighted in red) and a proximal tyrosine that may affect the motif's interaction potential upon phosphorylation [74], [75] (highlighted in cyan). The sequence pattern is conserved from *C. elegans* to human, but the tyrosine is conserved only between rat and human. This suggests that an old domain-binding motif has gained phospho-regulation in more recent organisms. Protein accessions are according to the Uniprot or Ensembl databases. (**B**) Potential phosphorylation site is older than the motif. The human Tau protein includes an SH3-binding motif (PxxP) and a proximal threonine that inhibits the motif's interaction potential upon phosphorylation [76]. This phosphorylation was also shown to induce a conformational change that unlocks the closed form of the protein [77]. The motif is conserved from *X. tropicalis* to human, while the potential phosphorylation site may have appeared earlier in evolution (present in *D. melanogaster*). This suggests that the domain-binding potential was established close to already functional phosphorylation.

**Figure 5:**
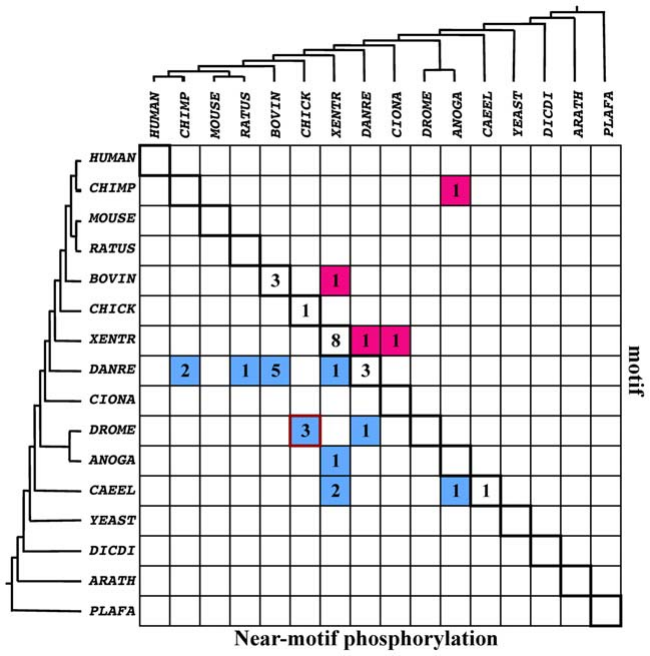
Phylogenetic traces of PDZ interaction-regulation unit evolution. This matrix summarizes the results for units of PDZ binding motifs and near-motif phosphorylation. The eukaryotic evolutionary tree is depicted above and left to the matrix (abbreviations below). The rows indicate the organism in which the motif probably appeared. The columns indicate the organism in which a potentially phosphorylated residue appeared. The order in which the motif and potentially phosphorylated residue appeared can thus be deduced from the matrix cells. For instance, the brown-framed cell represents the three cases in which the motif appeared in *D. melanogaster* and the potentially phosphorylated residue appeared in chicken. Accordingly, all cells below the diagonal (cyan) represent cases in which the potentially phosphorylated residue appeared after the motif. The diagonal cells represent cases in which the motif and the potentially phosphorylated residue appeared together. The cells above the diagonal represent cases in which the motif appeared after the potentially phosphorylated residue (red). Organism abbreviations: CHIMP- *p. troglodytes*, MOUSE- *m. musculus*, RATUS- *r. norvegicus*, BOVIN- *b. taurus*, CHICK- *g. gallus*, XENTR- *x. tropicalis*, DANRE- *d. rerio*, CIONA- *c. intestinalis*, DROME- *d. melanogaster*, ANOGA- *a. gambiae*, CAEEL- *c. elegans*, YEAST- *s. cerevisiae*, DICDI- *d. discoideum*, ARATH- *a. thaliana* and PLAFA- *p. falciparum*.

**Figure 6:**
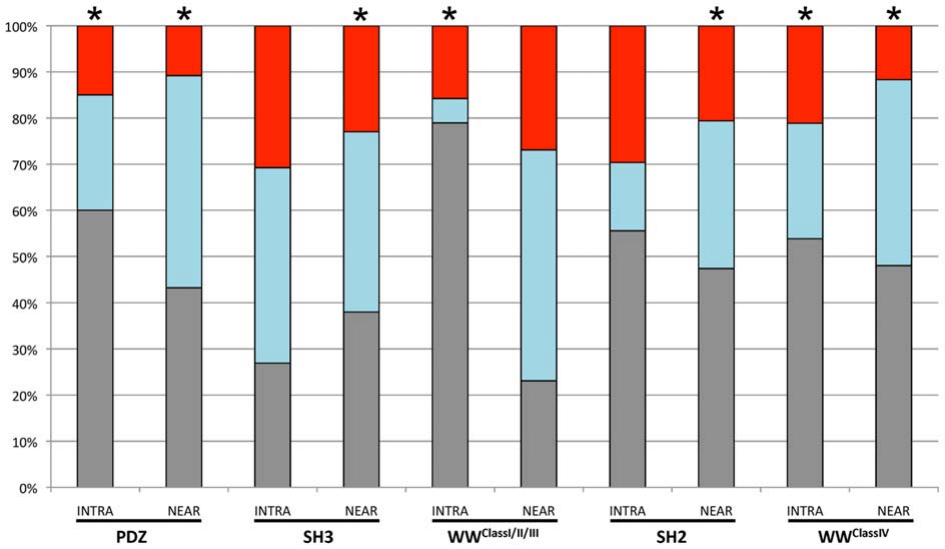
Frequency of various phylogenetic traces of motif-phosphorylation coupling. The stacked-bar graph details the relative frequency of the three possible phylogenetic traces of the interaction-regulation units (for either intra-motif phosphorylation or near-motif phosphorylation sites): (i) co-appearance of the motif and the potentially phosphorylated residue in the same organism (grey), (ii) the motif appeared before the potentially phosphorylated residue (cyan) (iii) the potentially phosphorylated residue appeared before the motif (red). For each domain we tested if the distribution of the various scenarios deviates from random by a χ^2^ test. Asterisks denote statistically significant results (based on Table S6).

Our comparative analysis, applied to an established eukaryotic phylogenetic tree [33], [34], suggested the oldest ancestor for each of the interaction-regulation unit components (see Methods). This allowed us to define three alternative evolutionary traces for the interaction-regulation unit evolution: (a) The motif and phosphorylation site appeared together in the same ancestor (b) the motif probably appeared before the phosphorylation site (exemplified in Figure 4A), and (c) the phosphorylation site probably appeared before the motif (exemplified in Figure 4B). As an example we show the results of evolutionary analysis of the coupling between PDZ-binding motif and near-motif phosphorylation in Figure 5. The evolutionary paths of interaction-regulation units for SH3-, PDZ- and class I/II/III WW-binding motifs, and the SH2- and class IV WW-binding motifs are detailed in Figure S2 and summarized in Figure 6. These results show that in many cases, the frequencies of the three possible evolutionary traces differ statistically significantly from a random model (p values range from 2e-7 for Class I/II/III WW motifs to 0.016 for PDZ domains, χ^2^ test, see Methods and Table S6). The different domain types are characterized by different frequencies of the possible paths (Figure 6). The trends are highly similar between the phospho-binding domains (SH2 and Class IV WW). The most common evolutionary trace for all domain-motif interaction types is the co-appearance of the interaction-regulation unit components (46% of all traces).

Of note, it is possible that the motif's sequence pattern includes a Ser/Thr/Tyr residue (for SH3/PDZ/Class I/II/III WW motifs) that is phosphorylated in human, leading to trivial co-appearance of the interaction-regulation unit components. To circumvent this potential bias, we repeated the analysis using only ELM-based regular expressions [14] that do not include Ser/Thr/Tyr as a means to locate motif hits in orthologs. We chose this motif pattern resource because this is the only repository in which the precise identity of all amino-acids is available (all other predictors note only one important residue within the motif). The over-abundance of co-appearing unit components stayed statistically significant. Furthermore, the co-appearance of the unit components is most frequently found in *X. Tropicalis* (see Figure S2). Thus, many motif-phosphorylation units have probably emerged after the vertebrate lineage has appeared, and are conserved from *X. tropicalis* to human.

## Discussion

Domain-motif interactions are instrumental for many central cellular processes, and are therefore tightly regulated. Phosphorylation events are known modulators of protein-protein interactions in general, including domain-motif interactions. The association between domain-motif interactions and phosphorylation events may stem from their similar interaction time scales (kinase-substrate interactions are themselves domain-motif interactions). Phospho-regulation of domain-motif interaction is apparent in cases where the motif-binding cleft in the domain is phosphorylated, resulting in loss of its interaction potential (for example SH3 [35], WW [36], PDZ [37], and SH2 domains [38]). Here, we addressed the association of phosphorylation and domain-motif interaction taking a motif-centred view. We integrated human domain-motif interaction and phosphorylation data for four representative domains (SH2, WW, SH3 and PDZ), and showed that their proximity and functional interrelationship may be more extensive than the previously established phospho-switching of phospho-binding domains (such as SH2 and class IV WW domains). Assuming that these four domain-motif interaction types are reliable representatives of such interactions, our results hint at the existence of unified units comprising motifs and associated phosphorylation sites, in which the regulation of domain-motif interaction is inherent.

### The manifold faces of phosphorylation as a switch

Our results expand the common phosphorylation-dependent ‘on/off’ switch of interaction by introducing ‘double switches’, where a phosphorylation event allows one interaction while concurrently preventing another interaction. The double switches described by us generalize similar sporadic cases, such as the one documented for Ataxin-1. There, phosphorylation and de-phosphorylation of a dual motif in Ataxin-1 permit its binding to splicing factors and to proteins of the 14-3-3 family, respectively [39].

The first kind of double switches we describe regards phosphorylation/de-phosphorylation of a stretch of residues that alternate its binding affinity to two different domains. Using strict patterns of SH2, SH3 and class I WW domains we identified 57 SH2-SH3 and SH2-WW dual motifs, for 16 of which we found supporting evidence in the human interactome, where three of those were indeed shown experimentally to function as double switches.

One intriguing candidate for SH3/SH2 phospho-switching is a dual motif, _567_PYLP_570_, present in the ABL2 kinase (it obeys the canonical PxxP SH3-binding motif and the Y[VLTFIC] Stat5 SH2-binding motif). This dual motif may suggest an alternative ABL2 regulation via on/off switching of self-interaction with its own SH3 and SH2 domains by Tyr-568 phosphorylation. Several lines of evidence strengthen this hypothesis: First, the corresponding tyrosine in mouse ABL-2 is documented as being phosphorylated [40]. Second, this segment is disordered according to IUPRED [41]. Finally, this segment is conserved throughout the eukaryotes, and is embedded within a less conserved context. The structural models of ABL-2 kinase [42] and the location of this segment, proximal, but outside the boundaries of the kinase domain, may suggest a model according to which Y568 phosphorylation is involved in switching the auto-inhibition of this kinase, or participate in the kinase mode that recognizes substrates.

Our results strengthen phosphorylation/de-phosphorylation double switch of protein-protein interactions as an important control mechanism among other widespread regulation schemes, mostly related to the cellular context of the proteins (for instance, protein expression/localization may determine which of two competing domains will bind a single motif). Cellular context may also affect phospho-switching of domain-motif interactions (*e.g.* through kinase/phosphatase levels).

To identify double switches between proteins of the same domain-family we compared the domain interaction pattern of peptides and pseudo-phosphorylated peptides using experimental PDZ-peptide interaction affinity values. Our results suggest that a single protein segment may bind two PDZ domains in a mutually exclusive manner, depending on its phosphorylation status. The three experimentally verified cases [21], [22], [23], along with the 81 potential double switches identified here, support the biological relevance of such double switches. PDZ domains are frequently found to be localized in the intracellular segment of membrane proteins. Many of these PDZ-containing proteins participate in key signaling complexes in the post synaptic density and are known to interact with the same targets [43]. Interestingly, 12 different proteins that are involved in nine putative double switches are associated with the post-synaptic density (Table S4).

Concurrent enabling and prevention of interaction involving domains from the same family may be static rather than temporal. For example, following our paper [44], we discovered a protein loop in the trypsin inhibitor domain that concurrently prevents homodimerization of trypsin/factor XIIA inhibitor while mediating its heterodimerization with alpha amylase via the same interface. Taken together, this indicates that functional protein traits (*e.g.* a structural element or phosphorylation), should be investigated for both their positive and negative effects, such as enhancement or prevention of interactions.

Our analysis revealed also a tight coupling between motifs and phosphorylation sites in their flanking 20 residues in both sides. This context length is in accord with previous publications studying the same domain-motif interaction types [8], [13]. In these papers, the authors showed that traits like disorder and co-evolving residues characterize segments of 15–20 residues flanking the motifs. The motif's context was previously shown to be an important determinant of domain-motif interaction specificity [8], both for domains that require and domains that do not require motif phosphorylation for binding [7]. Phosphorylation of the motif's context lends further support to the functional interrelationships between the motif and its context.

Phosphorylated residues near a domain-binding motif may affect the domain binding, as shown for the interaction between nuclear localization signal motifs and ARM domains [45]. Still, the proximity between motifs and phosphorylation sites might be co-incidental. A given motif may incidentally reside within a region including multiple phosphorylation sites, each of which is bound by a domain that binds phosphorylated motifs [46]. Alternatively, a motif can be bound by a protein that includes at least two domains: the motif-binding domain and a kinase domain. Consequently, the domain-motif interaction initiates a phosphorylation event near the motif (as exemplified for SH2 and SH3-binding motifs [47], [48], [49]). In order to have an approximation for the proportion of these scenarios, we tried to estimate the number of cases in which an SH2 domain is bound to a near-SH2 motif phosphorylation site. Using our experimentally-based domain-motif interaction database we found that 8% of these sites are themselves bound by SH2 domains. In nine cases (18%), the SH2-containing protein that binds this motif is a kinase. Very few examples of SH2-binding to near-motif phosphorylation sites were found for interaction-regulation units involving SH3/PDZ/WW binding motifs. The relative scarcity of these cases supports the interpretation of tyrosine phosphorylation near SH2 motifs as means to regulate SH2 binding by enhancing the availability of a kinase that will phosphorylate the nearby tyrosine, present in the SH2 motif. Enhancement of phosphorylation of the SH2-binding motif by nearby phosphorylation events is also supported by the high incidence of phospho-tyrosine in the vicinity of the motif.

The protein segment that includes the interaction-regulation unit is bound by multiple proteins: the kinase, the corresponding domain, and, in case of a double switch, a second domain. Thus, this protein region needs to adopt different conformations upon interaction with two or three distinct domains. This flexibility is probably feasible due to the tendency of phosphorylation sites and short motifs to reside in disordered regions ([13], [50], see Table S7). Protein disorder permits structural changes upon binding to different partners [51]. Furthermore, the disordered nature of the motif's context may allow the appropriate positioning against the binding domain [52]. The function of the disordered context suggests that changing this region (*e.g.* phosphorylation event) may affect the motif's binding-related behaviour.

### Evolutionary traces of motif-phosphorylation interaction-regulation units

We conducted sequence comparison between human and several eukaryotic organisms to trace the path in which the interaction-regulation unit components appeared during evolution. In 46% of the cases, the unit's components probably appeared together, frequently in vertebrates, and remained conserved along this lineage. Possibly, the co-appearance of the interaction-regulation unit components may trivially result from a protein that appeared in a certain phylogenetic branch and remained highly conserved up to human. Notably, in 77% of the cases where the unit components co-appeared, the protein that includes the unit has an ortholog in more distant organisms, but their sequence does not include the corresponding unit's region. This implies that, in general, the motif appears in evolution along with the potential to be phospho-regulated. This also agrees with the results of Chica *et al.* [53] who found that domain-binding motifs are typically conserved along the vertebrate lineage.

We identified also step-wise paths for the appearance of the interaction-regulation unit components. First, there are cases in which the motif probably appeared without being phospho-regulated. Such regulation appeared later, perhaps in cases where the domain-motif interaction required tighter regulation. The second evolutionary trace regards an early appearance of the potential phosphorylation site, followed by the motif's appearance. This scenario might be explained by an ancient functional phosphorylation event that has the potential to induce a conformational switch that exposes its nearby protein environment. This switch was later ‘hijacked’ by a newly introduced domain-binding motif that exploited it for its own regulation. The ‘early phosphorylation’ and the ‘early motif’ evolutionary traces cover 20% and 34%, respectively, out of the 481 studied evolutionary traces.

Conservation of phosphorylation was recently studied by Tan *et al.* [54] who suggested that there is a ‘core set’ of highly-conserved (yeast to human) phosphorylation sites, while other phosphorylation events evolve rapidly. The overlap between this ‘core set’ and the phosphorylation events in our suggested interaction-regulation units is negligible. This means that motif or near-motif phosphorylated residues are relatively recent (64% of the studied units include a potential phosphorylation site that appeared in vertebrates). Indeed, the modest requirement for 2–3 residues that are important for binding kinases (or domains) suggests that these phosphorylation sites and motifs appear and disappear in fast evolutionary rates, and have thus been suggested as fast-evolving agents of protein-protein interaction [1]. Moreover, the most plausible mechanism for the appearance of motifs and phosphorylation sites (that are in fact motifs bound by kinases) is convergent evolution. This may be advantageous for the evolution of protein regulatory regions and agrees with the recent proposal of disordered regions as significant contributors to the evolvability of proteins [55].

### Conclusions

Our findings have important implications for elucidating the function of motifs and phosphorylation events. The abundance of motif phospho-regulation implies that the search for novel domain-binding motifs should be followed by searching for intra/near phosphorylation sites. Similarly, newly discovered phosphorylation sites should be checked for a nearby binding motif, which may shed light on their function. The evolutionary trace of the motif and its flanking regions should assist in this regard. Another promising direction is towards a comprehensive protein-protein interaction network connecting between the interaction-regulation units, their corresponding kinases and the domain-containing proteins interacting with them. Analyzing this network should reveal novel associations between kinases and domain-motif interactions.

## Methods

### Human phosphorylation database

We integrated six databases of human experimentally-verified protein phosphorylation sites (Phospho.ELM [56], PhosphoSite [57], Uniprot [58], ProteinPedia [59], PHOSIDA [60] and HPRD [61]) and data from three additional phospho-proteomic surveys ([62], [63], [64]). To unify these databases and merge cases in which the same phosphorylation event is reported, we mapped all protein accession numbers to Uniprot accessions. To further verify the conversion, we kept only entries in which the protein sequence from each of the above databases is identical to the one of the corresponding Uniprot protein. In cases where different databases reported the same phosphorylation event but with different experimental methods – the evidences were unified. The resulting database was divided into two datasets depending on the source of phosphorylation event: (a) ‘low throughput’, highly reliable data based on low-throughput experiments (*e.g.* phospho-specific antibodies), and (b) ‘high throughput’, based on large-scale experiments (mostly mass-spectrometry).

### Human domain-motif interaction database

We extracted human domain-motif interactions from seven databases (PepCyber [65], Uniprot [58], DOMINO [66], ELM [14], PDZbase [67] and 3DID [68]) and integrated them into one unified catalogue of 2,683 such interactions. First, we filtered the database to include only interactions with motifs that are 30 residues or shorter. We chose this threshold to avoid the loss of experimentally-verified motifs that were not narrowed down by the experimentalists to the minimum length required for interaction (note that the average size of the motifs we used is 14 residues). To avoid redundancy originating from records of the same domain-motif interaction in multiple databases, we extracted all cases in which protein A (that includes a domain) and protein B (that includes a motif) are documented as interacting by two (or more) databases. If the motif boundaries were not overlapping according to the two databases – we kept both domain-motif interactions. In cases where the two databases reported motifs that overlap in >80% of the residues (*e.g.* the motif's resides in positions 30–42 according to the first database, and in positions 32–43 according to the second database), we chose only one domain-motif interaction (preferably the shorter motif). We focused on SH2, SH3, PDZ and WW domain-motif interactions, for which the largest amounts of data were available. This catalogue of 1,983 interactions was divided into two datasets: (a) ‘low throughput’, highly reliable data including 867 interactions based on low throughput experiments (*e.g.* mutagenesis or x-ray crystallography). (b) ‘high throughput’, including 1,116 interactions based on large scale experiments (*e.g.* protein micro-arrays). To compute the number of experimentally verified motifs that include a previously characterized sequence pattern, we used several repositories of such sequence patterns: Scansite [69], NetPhorest [4] and regular expressions derived from the ELM database [14].

### Statistical significance of intra/near motif phosphorylation events

To assess the statistical significance of our findings we compared the motif-phosphorylation coupling found in the data to that found in randomized datasets. For each protein that includes a motif of a certain type, we selected randomly same-length sub-sequences along the protein sequence while keeping the phosphorylation positions fixed. These random sequences obeyed the following constraints: (a) The random motif did not overlap with the actual motif and included a Ser/Thr/Tyr residue. (b) Since motifs and phosphorylated residues are known to reside within disordered protein regions [13], [50], we chose random motifs within regions predicted to be disordered at the same level as in the actual regions where the motifs reside (using the IUPred algorithm [41], see Table S7). For each set of proteins containing certain domain-binding motifs we repeated this procedure 10,000 times and counted the number of intra/near phosphorylation events. The fraction of random sets with counts that exceeded the count in the original data provides the statistical significance. The statistical significance values were corrected for multiple testing using Bonferroni correction.

### Compilation of data of domain-motif interactions and phosphorylation in *S. cerevisiae*

To create a database of motifs and phosphorylation sites in *S. cerevisiae*, we integrated high-throughput domain-motif interactions from Landgraf *et al.* [17], as well as phosphorylation data from the PhosphoGRID database [70]. We used the same filtering criteria as in the human database.

### Phosphorylation as a PDZ specificity switch

We used PDZ-peptide interaction affinity values derived from recently published peptide-array results [24], [25]. Peptide sequence similarity was computed using two substitution matrices: PAM30 [71] and a biophysical residue property matrix [72]. Aligned residues closer to the protein C-terminus, known as most important for PDZ-binding, were assigned with a higher weight [5]. We considered highly similar peptide couples where one peptide included Ser/Thr/Tyr (‘non-phosphorylated’) and the other included Asp/Glu in the corresponding position (‘pseudo-phosphorylated’). Notably, we kept only peptide pairs that had one identical and one highly similar aligned position within the last three residues of the peptides. We repeated this analysis for phosphorylated and pseudo-phosphorylated sites that reside in positions (-1), (-2) and (-3) from the C-terminus. This has yielded a list of all available pairs of non-phosphorylated and ‘pseudo-phosphorylated’ peptides. For each of these pairs, we checked whether there is a pair of PDZ domain proteins in the data, such that PDZ^a^ interacts with a non-phosphorylated peptide, but not with the ‘pseudo-phosphorylated’ peptide, and PDZ^b^ displays an inverse binding pattern. We identified 81 peptide-PDZ double pairs that showed this binding pattern (60 for position -1, one for position -2 and 20 for position -3). These PDZ pairs were used to evaluate the statistical significance of the results. In principle, given a non-phosphorylated/pseudo-phosphorylated peptide pair and two PDZ domains there are 10 possible scenarios of binding/non-binding relationships among them (Table S5), with the double switch being one of these possibilities. The count of the double switch scenario and the total count of all other binding scenarios were compared to the respective counts expected at random by Fisher's exact test. The over-representation of the double switch was statistically significant for peptides that were non-phosphorylated/‘pseudo-phosphorylated’ in positions (-1) and (-3) (p≤0.0003 and p≤0.0031, respectively, see Table S5).

### Evolution of motif-phosphorylation coupling

The set of human motifs that were used for the evolutionary analysis was restricted to motifs that obey a previously characterized sequence pattern. For SH3, SH2 and PDZ motifs, we used ScanSite prediction [69]. For WW class I/II/III motifs, we used patterns from the ELM database. For Class IV WW motifs, we used Netphorest predictions (here, we verified that the relevant Ser/Thr are phosphorylated according to our phosphorylation database). To catalogue orthologs for each of the human interaction-regulation units, we used the Inparanoid database. Additional orthologs were added using best reciprocal BLAST hits between the human protein and any given eukaryotic species (e-value threshold for BLAST comparisons was 1e-6; protein sequences were taken from the Uniprot and NCBI databases [34], [58]). Notably, all four domains, as defined by the Pfam database [73], were found to be present in the proteomes of all the 15 model organisms (with the exception of SH3 and SH2 domains in *P. falciparum)*. The conservation level of human motifs and phosphorylation sites were deduced from pair-wise sequence alignment between the human protein and each relevant ortholog. We chose a pair-wise, rather than multiple sequence alignment approach, since the multiple sequence alignment scheme produced mis-aligned segments of ortholog sequences. This was probably due to the fact that the differences between the orthologs were not consistent (in terms of substitutions and insertions/deletions). The organism that is most distant from human and has a Ser/Thr/Tyr residue in a corresponding position was set as the organism in which the potential phosphorylation site first appeared. Likewise, we determined the organism in which the motif appeared. Here, we searched for a respective sequence pattern hit by the four servers mentioned above. Since the location of motifs in orthologs was previously shown to be flexible, we considered motif appearance if it was found within 20 residues range (N/C-terminal) of the sequence that aligned with the human motif. Note that the different pattern recognition tools identify different subtypes of the motif. We unified these different types and treated them as one. In the evolutionary analysis of the SH2- and class IV WW-motifs, we disregarded the phosphorylation sites that are essential for the interaction. For this analysis we used both the low throughput and the high throughput datasets of the human domain-motif interactions and phosphorylation events.

To assess the statistical significance of the over-representation of the co-appearance of the motif and potential phosphorylation site over stepwise appearance of the interaction-regulation unit components, we used the following approach, repeated for each domain type. For each organism we calculated the frequency in which motifs appeared first in this specific organism, and the frequency in which a potential phosphorylation site appeared first in this organism. For each possible organism-organism comparison, we multiplied these two frequencies to get the expected frequency for the appearance of the motif and the phosphorylation site for this organism combination. Similarly, we calculated the expected frequency of step-wise appearance of the motif and potential phosphorylation site. Summation over all organism-organism combinations provided the expected fractions for co-appearance and step-wise appearances, respectively (Table S6). The statistical significance of the results for each domain was obtained by comparison between the actual counts and those expected at random by a χ^2^ test (Table S6). The statistical significance values were corrected for multiple testing (over the various domains) using Bonferroni correction.

## Supporting Information

Figure S1 **The disordered contexts of the motifs.**
(PDF)

Figure S2 **Phylogenetic traces of interaction-regulation unit evolution.**
(PDF)

Table S1 **Counts and statistical significance of the coupling between domain-motif interactions and phosphorylations.**
(PDF)

Table S2 **Identification of human protein segments that display a dual motif that can act as a phosphorylation double switch for SH3 and SH2 domains or for SH2 and WW domains.**
(PDF)

Table S3 **Proteins that are predicted to be involved in ‘double switches’ based on experimental protein-protein interaction data.**
(PDF)

Table S4 **Phosphorylation specificity switch for proteins containing domains of the PDZ family.**
(PDF)

Table S5 **Statistical significance for the PDZ double-switch analysis.**
(PDF)

Table S6 **Statistical significance for the evolutionary path analysis.**
(PDF)

Table S7 **Disorder levels of the various motifs.**
(PDF)

Dataset S1 **Database of interaction-regulation units for** ***Homo sapiens*** **.**
(XLS)

Dataset S2 **Database of interaction-regulation units for** ***Saccharomyces cerevisiae***.
(XLS)

## References

[1] Neduva V, Russell RB (2005). Linear motifs: evolutionary interaction switches.. FEBS Lett.

[2] Pawson T, Raina M, Nash P (2002). Interaction domains: from simple binding events to complex cellular behavior.. FEBS Lett.

[3] Kaneko T, Li L, Li SS (2008). The SH3 domain–a family of versatile peptide- and protein-recognition module.. Front Biosci.

[4] Miller ML, Jensen LJ, Diella F, Jorgensen C, Tinti M (2008). Linear motif atlas for phosphorylation-dependent signaling.. Sci Signal.

[5] Nourry C, Grant SG, Borg JP (2003). PDZ domain proteins: plug and play!. Sci STKE.

[6] Chica C, Diella F, Gibson TJ (2009). Evidence for the concerted evolution between short linear protein motifs and their flanking regions.. PLoS One.

[7] Seet BT, Dikic I, Zhou MM, Pawson T (2006). Reading protein modifications with interaction domains.. Nat Rev Mol Cell Biol.

[8] Stein A, Aloy P (2008). Contextual specificity in peptide-mediated protein interactions.. PLoS One.

[9] Narayanan A, Jacobson MP (2009). Computational studies of protein regulation by post-translational phosphorylation.. Curr Opin Struct Biol.

[10] Johnson LN, Barford D (1993). The effects of phosphorylation on the structure and function of proteins.. Annu Rev Biophys Biomol Struct.

[11] Yaffe MB, Elia AE (2001). Phosphoserine/threonine-binding domains.. Curr Opin Cell Biol.

[12] Zhou GL, Zhuo Y, King CC, Fryer BH, Bokoch GM (2003). Akt phosphorylation of serine 21 on Pak1 modulates Nck binding and cell migration.. Mol Cell Biol.

[13] Fuxreiter M, Tompa P, Simon I (2007). Local structural disorder imparts plasticity on linear motifs.. Bioinformatics.

[14] Gould CM, Diella F, Via A, Puntervoll P, Gemund C (2010). ELM: the status of the 2010 eukaryotic linear motif resource.. Nucleic Acids Res.

[15] James M, Nuttall A, Ilsley JL, Ottersbach K, Tinsley JM (2000). Adhesion-dependent tyrosine phosphorylation of (beta)-dystroglycan regulates its interaction with utrophin.. J Cell Sci.

[16] Birrane G, Chung J, Ladias JA (2003). Novel mode of ligand recognition by the Erbin PDZ domain.. J Biol Chem.

[17] Landgraf C, Panni S, Montecchi-Palazzi L, Castagnoli L, Schneider-Mergener J (2004). Protein interaction networks by proteome peptide scanning.. PLoS Biol.

[18] Ceol A, Chatr Aryamontri A, Licata L, Peluso D, Briganti L (2010). MINT, the molecular interaction database: 2009 update.. Nucleic Acids Res.

[19] Aranda B, Achuthan P, Alam-Faruque Y, Armean I, Bridge A (2010). The IntAct molecular interaction database in 2010.. Nucleic Acids Res.

[20] Salwinski L, Miller CS, Smith AJ, Pettit FK, Bowie JU (2004). The Database of Interacting Proteins: 2004 update.. Nucleic Acids Res.

[21] Adey NB, Huang L, Ormonde PA, Baumgard ML, Pero R (2000). Threonine phosphorylation of the MMAC1/PTEN PDZ binding domain both inhibits and stimulates PDZ binding.. Cancer Res.

[22] Hegedus T, Sessler T, Scott R, Thelin W, Bakos E (2003). C-terminal phosphorylation of MRP2 modulates its interaction with PDZ proteins.. Biochem Biophys Res Commun.

[23] von Nandelstadh P, Ismail M, Gardin C, Suila H, Zara I (2009). A class III PDZ binding motif in the myotilin and FATZ families binds enigma family proteins: a common link for Z-disc myopathies.. Mol Cell Biol.

[24] Chen JR, Chang BH, Allen JE, Stiffler MA, MacBeath G (2008). Predicting PDZ domain-peptide interactions from primary sequences.. Nat Biotechnol.

[25] Stiffler MA, Chen JR, Grantcharova VP, Lei Y, Fuchs D (2007). PDZ domain binding selectivity is optimized across the mouse proteome.. Science.

[26] Choi J, Ko J, Park E, Lee JR, Yoon J (2002). Phosphorylation of stargazin by protein kinase A regulates its interaction with PSD-95.. J Biol Chem.

[27] Koo BK, Jung YS, Shin J, Han I, Mortier E (2006). Structural basis of syndecan-4 phosphorylation as a molecular switch to regulate signaling.. J Mol Biol.

[28] Parker LL, Backstrom JR, Sanders-Bush E, Shieh BH (2003). Agonist-induced phosphorylation of the serotonin 5-HT2C receptor regulates its interaction with multiple PDZ protein 1.. J Biol Chem.

[29] Trost M, English L, Lemieux S, Courcelles M, Desjardins M (2009). The phagosomal proteome in interferon-gamma-activated macrophages.. Immunity.

[30] Sonoda T, Mochizuki C, Yamashita T, Watanabe-Kaneko K, Miyagi Y (2006). Binding of glutamate receptor delta2 to its scaffold protein, Delphilin, is regulated by PKA.. Biochem Biophys Res Commun.

[31] Ostlund G, Schmitt T, Forslund K, Kostler T, Messina DN (2010). InParanoid 7: new algorithms and tools for eukaryotic orthology analysis.. Nucleic Acids Res.

[32] Gauci S, Helbig AO, Slijper M, Krijgsveld J, Heck AJ (2009). Lys-N and trypsin cover complementary parts of the phosphoproteome in a refined SCX-based approach.. Anal Chem.

[33] Letunic I, Bork P (2007). Interactive Tree Of Life (iTOL): an online tool for phylogenetic tree display and annotation.. Bioinformatics.

[34] Pruitt KD, Tatusova T, Maglott DR (2003). NCBI Reference Sequence project: update and current status.. Nucleic Acids Res.

[35] Wu Y, Spencer SD, Lasky LA (1998). Tyrosine phosphorylation regulates the SH3-mediated binding of the Wiskott-Aldrich syndrome protein to PSTPIP, a cytoskeletal-associated protein.. J Biol Chem.

[36] Lu PJ, Zhou XZ, Liou YC, Noel JP, Lu KP (2002). Critical role of WW domain phosphorylation in regulating phosphoserine binding activity and Pin1 function.. J Biol Chem.

[37] Gardoni F, Mauceri D, Fiorentini C, Bellone C, Missale C (2003). CaMKII-dependent phosphorylation regulates SAP97/NR2A interaction.. J Biol Chem.

[38] Couture C, Songyang Z, Jascur T, Williams S, Tailor P (1996). Regulation of the Lck SH2 domain by tyrosine phosphorylation.. J Biol Chem.

[39] de Chiara C, Menon RP, Strom M, Gibson TJ, Pastore A (2009). Phosphorylation of S776 and 14-3-3 binding modulate ataxin-1 interaction with splicing factors.. PLoS One.

[40] Tanis KQ, Veach D, Duewel HS, Bornmann WG, Koleske AJ (2003). Two distinct phosphorylation pathways have additive effects on Abl family kinase activation.. Mol Cell Biol.

[41] Dosztanyi Z, Csizmok V, Tompa P, Simon I (2005). IUPred: web server for the prediction of intrinsically unstructured regions of proteins based on estimated energy content.. Bioinformatics.

[42] Colicelli J (2010). ABL tyrosine kinases: evolution of function, regulation, and specificity.. Sci Signal.

[43] Feng W, Zhang M (2009). Organization and dynamics of PDZ-domain-related supramodules in the postsynaptic density.. Nat Rev Neurosci.

[44] Akiva E, Itzhaki Z, Margalit H (2008). Built-in loops allow versatility in domain-domain interactions: lessons from self-interacting domains.. Proc Natl Acad Sci U S A.

[45] Alvisi G, Rawlinson SM, Ghildyal R, Ripalti A, Jans DA (2008). Regulated nucleocytoplasmic trafficking of viral gene products: a therapeutic target?. Biochim Biophys Acta.

[46] Yang XJ (2005). Multisite protein modification and intramolecular signaling.. Oncogene.

[47] Filippakopoulos P, Kofler M, Hantschel O, Gish GD, Grebien F (2008). Structural coupling of SH2-kinase domains links Fes and Abl substrate recognition and kinase activation.. Cell.

[48] Pellicena P, Miller WT (2001). Processive phosphorylation of p130Cas by Src depends on SH3-polyproline interactions.. J Biol Chem.

[49] Summy JM, Guappone AC, Sudol M, Flynn DC (2000). The SH3 and SH2 domains are capable of directing specificity in protein interactions between the non-receptor tyrosine kinases cSrc and cYes.. Oncogene.

[50] Iakoucheva LM, Radivojac P, Brown CJ, O'Connor TR, Sikes JG (2004). The importance of intrinsic disorder for protein phosphorylation.. Nucleic Acids Res.

[51] Oldfield CJ, Meng J, Yang JY, Yang MQ, Uversky VN (2008). Flexible nets: disorder and induced fit in the associations of p53 and 14-3-3 with their partners.. BMC Genomics.

[52] Tompa P (2002). Intrinsically unstructured proteins.. Trends Biochem Sci.

[53] Chica C, Labarga A, Gould CM, Lopez R, Gibson TJ (2008). A tree-based conservation scoring method for short linear motifs in multiple alignments of protein sequences.. BMC Bioinformatics.

[54] Tan CS, Bodenmiller B, Pasculescu A, Jovanovic M, Hengartner MO (2009). Comparative analysis reveals conserved protein phosphorylation networks implicated in multiple diseases.. Sci Signal.

[55] Tokuriki N, Tawfik DS (2009). Protein dynamism and evolvability.. Science.

[56] Diella F, Gould CM, Chica C, Via A, Gibson TJ (2008). Phospho.ELM: a database of phosphorylation sites–update 2008.. Nucleic Acids Res.

[57] Hornbeck PV, Chabra I, Kornhauser JM, Skrzypek E, Zhang B (2004). PhosphoSite: A bioinformatics resource dedicated to physiological protein phosphorylation.. Proteomics.

[58] Bairoch A, Apweiler R, Wu CH, Barker WC, Boeckmann B (2005). The Universal Protein Resource (UniProt).. Nucleic Acids Res.

[59] Kandasamy K, Keerthikumar S, Goel R, Mathivanan S, Patankar N (2009). Human Proteinpedia: a unified discovery resource for proteomics research.. Nucleic Acids Res.

[60] Gnad F, Ren S, Cox J, Olsen JV, Macek B (2007). PHOSIDA (phosphorylation site database): management, structural and evolutionary investigation, and prediction of phosphosites.. Genome Biol.

[61] Keshava Prasad TS, Goel R, Kandasamy K, Keerthikumar S, Kumar S (2009). Human Protein Reference Database–2009 update.. Nucleic Acids Res.

[62] Linding R, Jensen LJ, Ostheimer GJ, van Vugt MA, Jorgensen C (2007). Systematic discovery of in vivo phosphorylation networks.. Cell.

[63] Old WM, Shabb JB, Houel S, Wang H, Couts KL (2009). Functional proteomics identifies targets of phosphorylation by B-Raf signaling in melanoma.. Mol Cell.

[64] Oppermann FS, Gnad F, Olsen JV, Hornberger R, Greff Z (2009). Large-scale proteomics analysis of the human kinome.. Mol Cell Proteomics.

[65] Gong W, Zhou D, Ren Y, Wang Y, Zuo Z (2008). PepCyber:P∼PEP: a database of human protein protein interactions mediated by phosphoprotein-binding domains.. Nucleic Acids Res.

[66] Ceol A, Chatr-aryamontri A, Santonico E, Sacco R, Castagnoli L (2007). DOMINO: a database of domain-peptide interactions.. Nucleic Acids Res.

[67] Beuming T, Skrabanek L, Niv MY, Mukherjee P, Weinstein H (2005). PDZBase: a protein-protein interaction database for PDZ-domains.. Bioinformatics.

[68] Stein A, Panjkovich A, Aloy P (2009). 3did Update: domain-domain and peptide-mediated interactions of known 3D structure.. Nucleic Acids Res.

[69] Obenauer JC, Cantley LC, Yaffe MB (2003). Scansite 2.0: Proteome-wide prediction of cell signaling interactions using short sequence motifs.. Nucleic Acids Res.

[70] Stark C, Su TC, Breitkreutz A, Lourenco P, Dahabieh M (2010). PhosphoGRID: a database of experimentally verified in vivo protein phosphorylation sites from the budding yeast Saccharomyces cerevisiae.. Database (Oxford).

[71] Gonnet GH, Cohen MA, Benner SA (1992). Exhaustive matching of the entire protein sequence database.. Science.

[72] Grantham R (1974). Amino acid difference formula to help explain protein evolution.. Science.

[73] Finn RD, Mistry J, Tate J, Coggill P, Heger A (2010). The Pfam protein families database.. Nucleic Acids Res.

[74] Chu I, Sun J, Arnaout A, Kahn H, Hanna W (2007). p27 phosphorylation by Src regulates inhibition of cyclin E-Cdk2.. Cell.

[75] Harkiolaki M, Tsirka T, Lewitzky M, Simister PC, Joshi D (2009). Distinct binding modes of two epitopes in Gab2 that interact with the SH3C domain of Grb2.. Structure.

[76] Reynolds CH, Garwood CJ, Wray S, Price C, Kellie S (2008). Phosphorylation regulates tau interactions with Src homology 3 domains of phosphatidylinositol 3-kinase, phospholipase Cgamma1, Grb2, and Src family kinases.. J Biol Chem.

[77] Lin YT, Cheng JT, Liang LC, Ko CY, Lo YK (2007). The binding and phosphorylation of Thr231 is critical for Tau's hyperphosphorylation and functional regulation by glycogen synthase kinase 3beta.. J Neurochem.

[78] Cohen NA, Brenman JE, Snyder SH, Bredt DS (1996). Binding of the inward rectifier K+ channel Kir 2.3 to PSD-95 is regulated by protein kinase A phosphorylation.. Neuron.

[79] Hu LA, Chen W, Premont RT, Cong M, Lefkowitz RJ (2002). G protein-coupled receptor kinase 5 regulates beta 1-adrenergic receptor association with PSD-95.. J Biol Chem.

[80] Tanemoto M, Fujita A, Higashi K, Kurachi Y (2002). PSD-95 mediates formation of a functional homomeric Kir5.1 channel in the brain.. Neuron.

[81] Cao TT, Deacon HW, Reczek D, Bretscher A, von Zastrow M (1999). A kinase-regulated PDZ-domain interaction controls endocytic sorting of the beta2-adrenergic receptor.. Nature.

[82] Sulka B, Lortat-Jacob H, Terreux R, Letourneur F, Rousselle P (2009). Tyrosine dephosphorylation of the syndecan-1 PDZ binding domain regulates syntenin-1 recruitment.. J Biol Chem.

[83] Boisguerin P, Ay B, Radziwill G, Fritz RD, Moelling K (2007). Characterization of a putative phosphorylation switch: adaptation of SPOT synthesis to analyze PDZ domain regulation mechanisms.. Chembiochem.

[84] Sengupta D, Linstedt AD (2010). Mitotic inhibition of GRASP65 organelle tethering involves Polo-like kinase 1 (PLK1) phosphorylation proximate to an internal PDZ ligand.. J Biol Chem.

[85] Anggono V, Smillie KJ, Graham ME, Valova VA, Cousin MA (2006). Syndapin I is the phosphorylation-regulated dynamin I partner in synaptic vesicle endocytosis.. Nat Neurosci.

[86] Anggono V, Robinson PJ (2007). Syndapin I and endophilin I bind overlapping proline-rich regions of dynamin I: role in synaptic vesicle endocytosis.. J Neurochem.

[87] Aragon E, Goerner N, Zaromytidou AI, Xi Q, Escobedo A (2011). A Smad action turnover switch operated by WW domain readers of a phosphoserine code.. Genes Dev.

[88] Kesti T, Ruppelt A, Wang JH, Liss M, Wagner R (2007). Reciprocal regulation of SH3 and SH2 domain binding via tyrosine phosphorylation of a common site in CD3epsilon.. J Immunol.

[89] Arevalo JC, Pereira DB, Yano H, Teng KK, Chao MV (2006). Identification of a switch in neurotrophin signaling by selective tyrosine phosphorylation.. J Biol Chem.

[90] Sotgia F, Lee H, Bedford MT, Petrucci T, Sudol M (2001). Tyrosine phosphorylation of beta-dystroglycan at its WW domain binding motif, PPxY, recruits SH2 domain containing proteins.. Biochemistry.

[91] Uyttendaele I, Lemmens I, Verhee A, De Smet AS, Vandekerckhove J (2007). Mammalian protein-protein interaction trap (MAPPIT) analysis of STAT5, CIS, and SOCS2 interactions with the growth hormone receptor.. Mol Endocrinol.

[92] Wang X, Darus CJ, Xu BC, Kopchick JJ (1996). Identification of growth hormone receptor (GHR) tyrosine residues required for GHR phosphorylation and JAK2 and STAT5 activation.. Mol Endocrinol.

[93] Wu C, Ma MH, Brown KR, Geisler M, Li L (2007). Systematic identification of SH3 domain-mediated human protein-protein interactions by peptide array target screening.. Proteomics.

[94] Deckert M, Elly C, Altman A, Liu YC (1998). Coordinated regulation of the tyrosine phosphorylation of Cbl by Fyn and Syk tyrosine kinases.. J Biol Chem.

[95] Sanjay A, Miyazaki T, Itzstein C, Purev E, Horne WC (2006). Identification and functional characterization of an Src homology domain 3 domain-binding site on Cbl.. Febs J.

