# Correction: A Dynamic View of Domain-Motif Interactions

**DOI:** 10.1371/annotation/2e21b1b9-46de-4cbe-a2a4-b4598d90d492

**Published:** 2012-01-23

**Authors:** Eyal Akiva, Gilgi Friedlander, Zohar Itzhaki, Hanah Margalit

The current addresses of Gilgi Friedlander and Zohar Itzhaki were incorrect. The correct current addresses are:

¤b Current address: Bioinformatics and Biological Computing Unit, Weizmann Institute of Science, Rehovot, Israel

¤c Current address: Department of Immunology, Weizmann Institute of Science, Rehovot, Israel 

