# Supplementary material for: A Dynamic View of Domain-Motif Interactions
Source: PLoS Comput Biol. 2012 Jan 12;8(1):e1002341. doi: 10.1371/journal.pcbi.1002341 (PMC3257277; doi:10.1371/journal.pcbi.1002341)
Supplement: Figure S1 — The disordered contexts of the motifs. (PDF) [file pcbi.1002341.s001.pdf]

Figure S1: The disordered contexts of the motifs

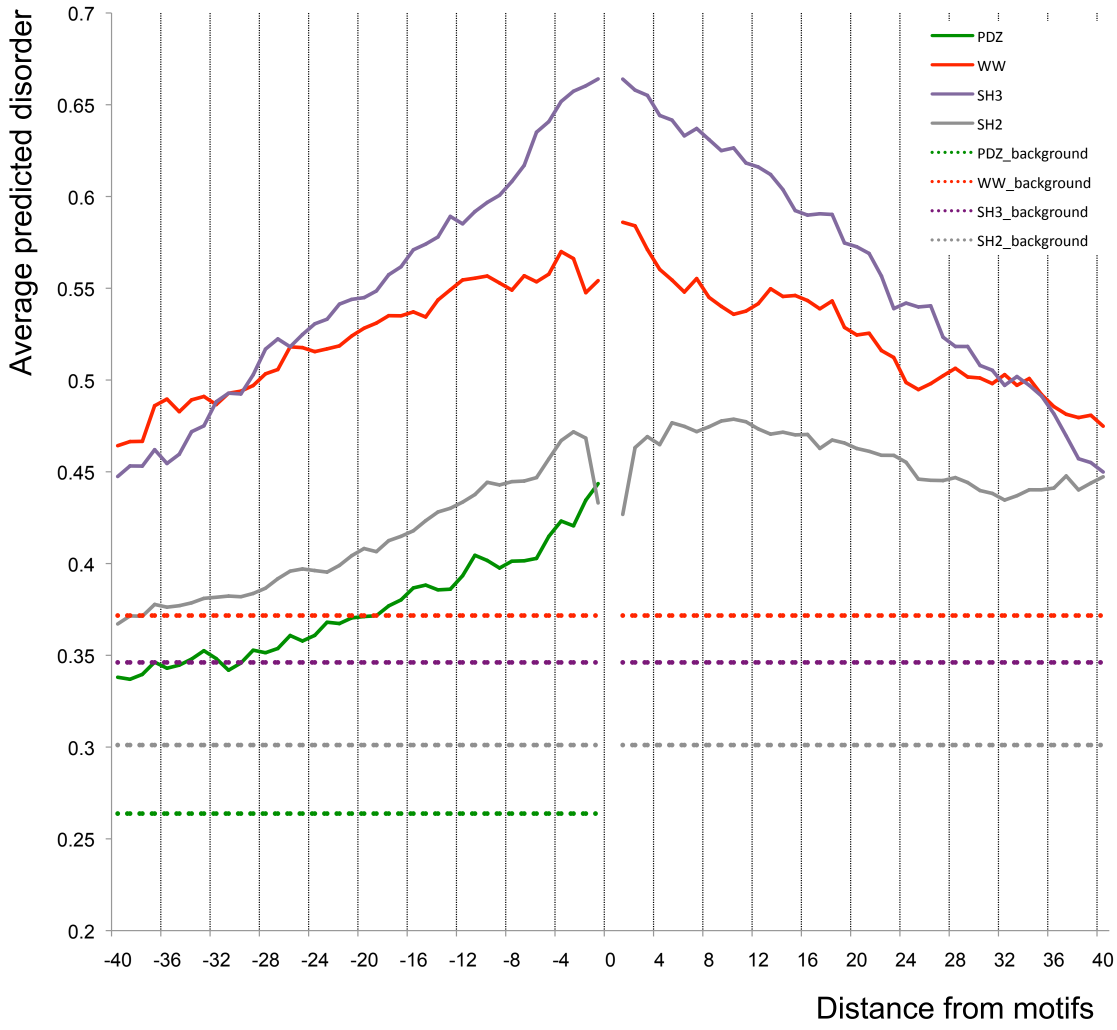

The graph demonstrates the increased predicted disorder in the flanking regions of PDZ, WW, SH3 and SH2 binding motifs. Proteins that include a certain motif type were assigned with predicted disorder (using IUPred, values range from 0 to 1 and are denoted on the y-axis) for each position. The 'background' disorder tendency was defined by the average disorder of the complete sequences of all proteins that include a certain motif (dashed lines). The x-axis denotes positions relative to each of the motifs. "0" indicates the motif's position (with no y-values). Positive and negative values represent positions that are N-terminal or C-terminal to the motifs, respectively. We took all proteins that include a certain motif, aligned them by the motif positions, and computed the average predicted disorder at each position (solid lines).
