# Supplementary material for: A Dynamic View of Domain-Motif Interactions
Source: PLoS Comput Biol. 2012 Jan 12;8(1):e1002341. doi: 10.1371/journal.pcbi.1002341 (PMC3257277; doi:10.1371/journal.pcbi.1002341)
Supplement: Figure S2 — Phylogenetic traces of interaction-regulation unit evolution. (PDF) [file pcbi.1002341.s002.pdf]

Figure S2: Phylogenetic traces of interaction-regulation unit evolution

Each figure part (A-E) includes two square matrices, the left summarizes the results for near-motif phosphorylation units, and the right summarizes the results for intra-motif phosphorylation units. The eukaryotic evolutionary tree is depicted on the left and upper sides of each square matrix. The rows indicate the organism in which the motif probably appeared. The columns indicate the organism in which a potentially phosphorylated residue appeared (this residue aligns with the phosphorylated amino acid in human). The order in which the motif and potentially phosphorylated residue appeared can thus be deduced from the table cells. For instance, the brown-framed value in (A) represents the three cases in which the motif appeared in D. melanogaster and the potentially phosphorylated residue appeared in chicken. Accordingly, all cells below the diagonal (cyan) represent cases in which the potentially phosphorylated residue appeared after the motif. The diagonal cells represent cases in which the motif and the potentially phosphorylated residue appeared together. The cells above the diagonal represent cases in which the motif appeared after the potentially phosphorylated residue (red). (A) PDZ units (B) SH3 units (C) Class I/II/III WW units (D) Class IV WW units (E) SH2 units.

Organism name abbreviations: CHIMP- *Pan troglodytes*, MOUSE- *Mus musculus*, RATUS- *Rattus norvegicus*, BOVIN- *Bos taurus*, CHICK- *Gallus gallus*, DANRE- *Danio rerio*, XENTR- *Xenopus tropicalis*, CIONA- *Ciona intestinalis*, DROME- *Drosophila melanogaster*, ANOGA- *Anopheles gambiae*, CAEEL- *Caenorhabditis elegans*, YEAST- *Saccharomyces cerevisiae*, DICDI- *Dictyostelium discoideum*, ARATH- *Arabidopsis thaliana* and PLAFA- *Plasmodium falciparum*.

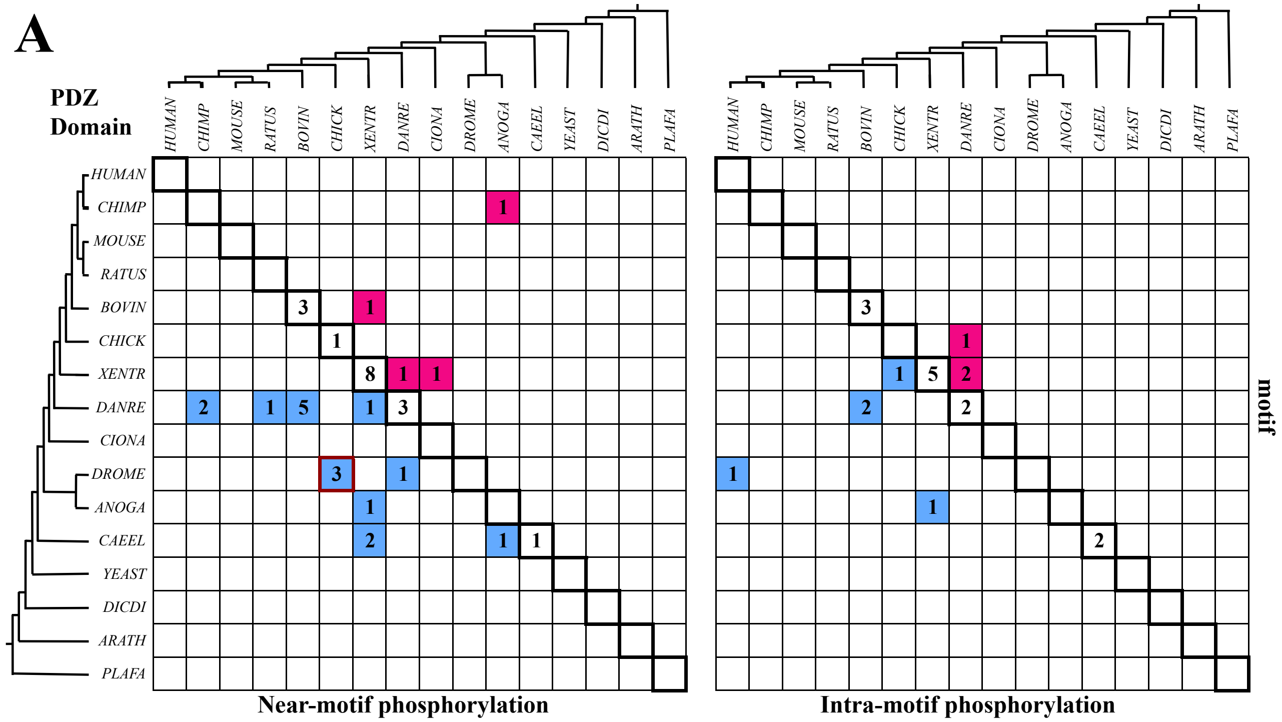

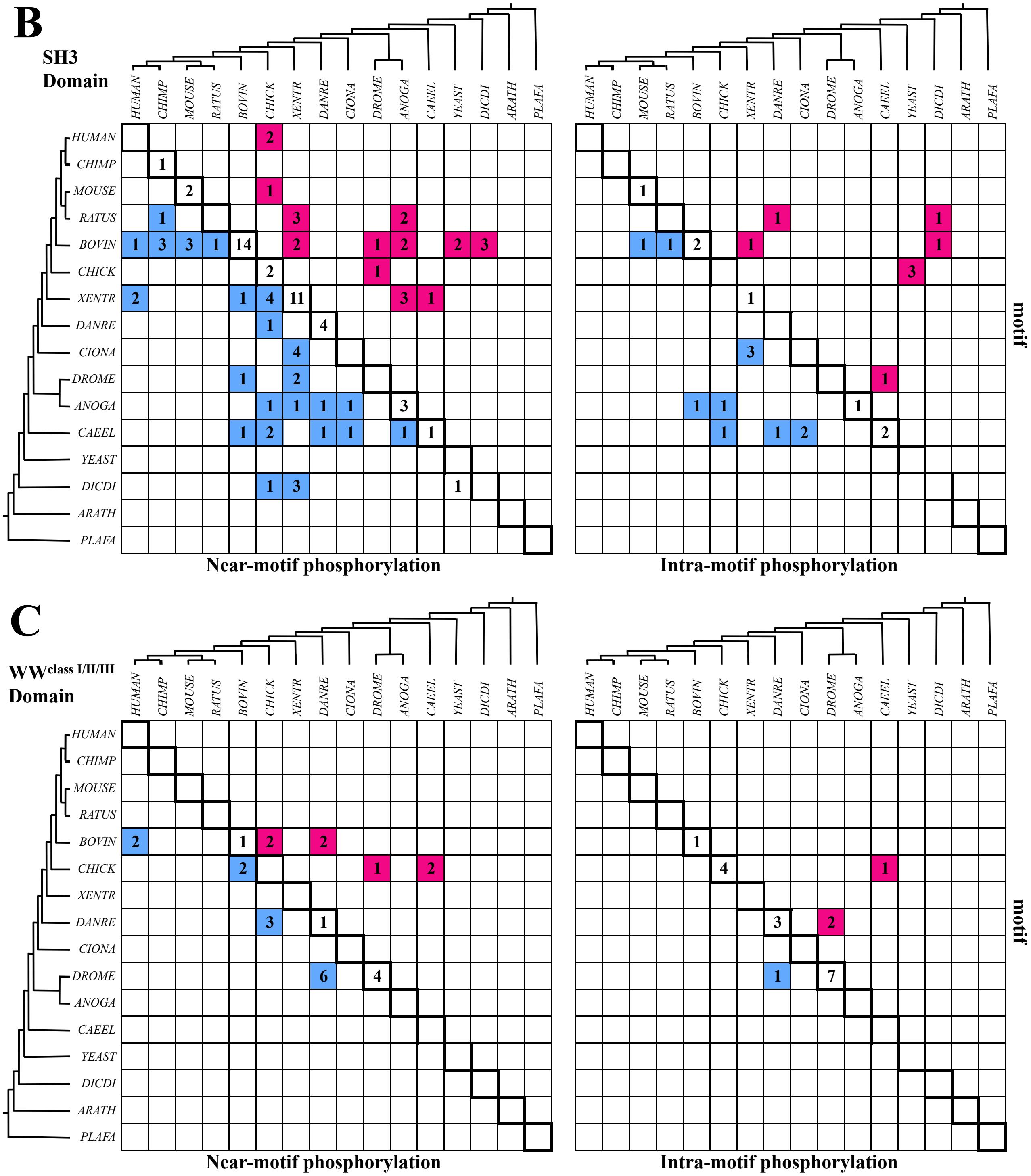
B
SH3
Domain
HUMAN CHIMP MOUSE RATUS BOVIN CHICK XENTR DANRE CIONA DROME ANOGA CAEEL YEAST DICDI ARATH PLAFA
Near-motif phosphorylation
Intra-motif phosphorylation
motif
C
WW class I/II/III
Domain
HUMAN CHIMP MOUSE RATUS BOVIN CHICK XENTR DANRE CIONA DROME ANOGA CAEEL YEAST DICDI ARATH PLAFA
Near-motif phosphorylation
Intra-motif phosphorylation
motif

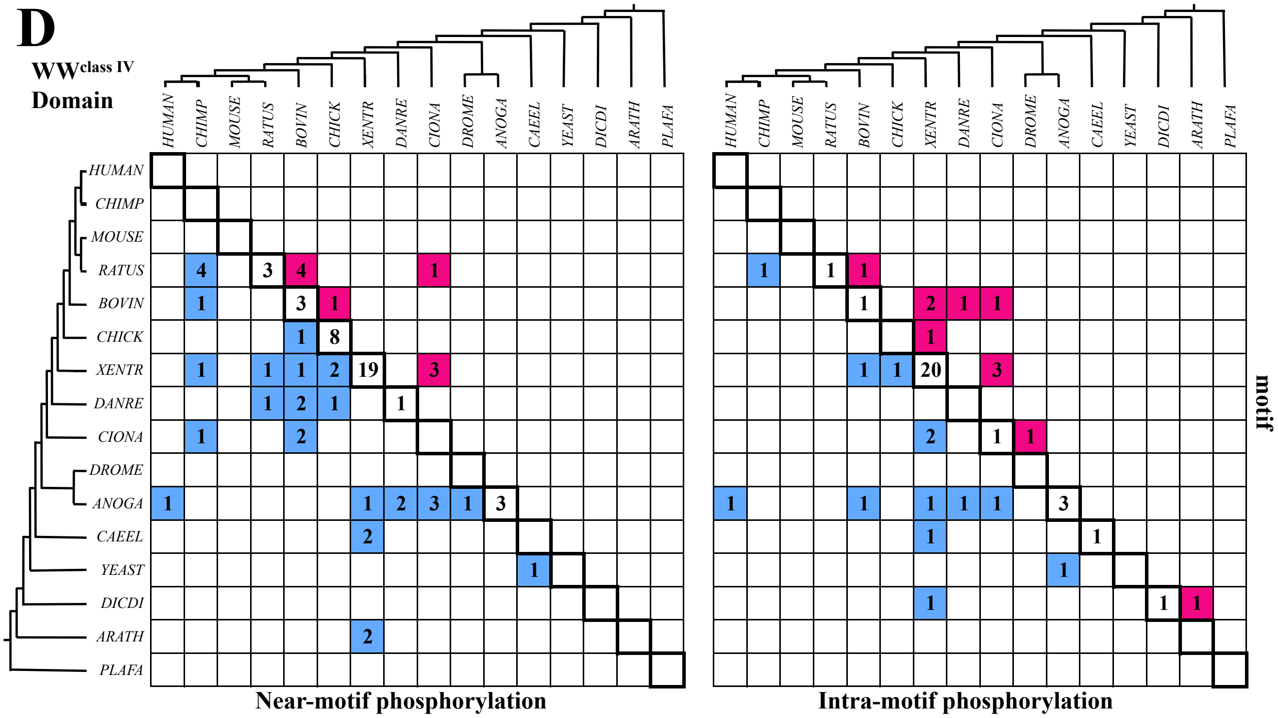
D
WW class IV Domain
HUMAN CHIMP MOUSE RATUS BOVIN CHICK XENTR DANRE CIONA DROME ANOGA CAEEL YEAST DICDI ARATH PLAFA
Near-motif phosphorylation
Intra-motif phosphorylation
motif

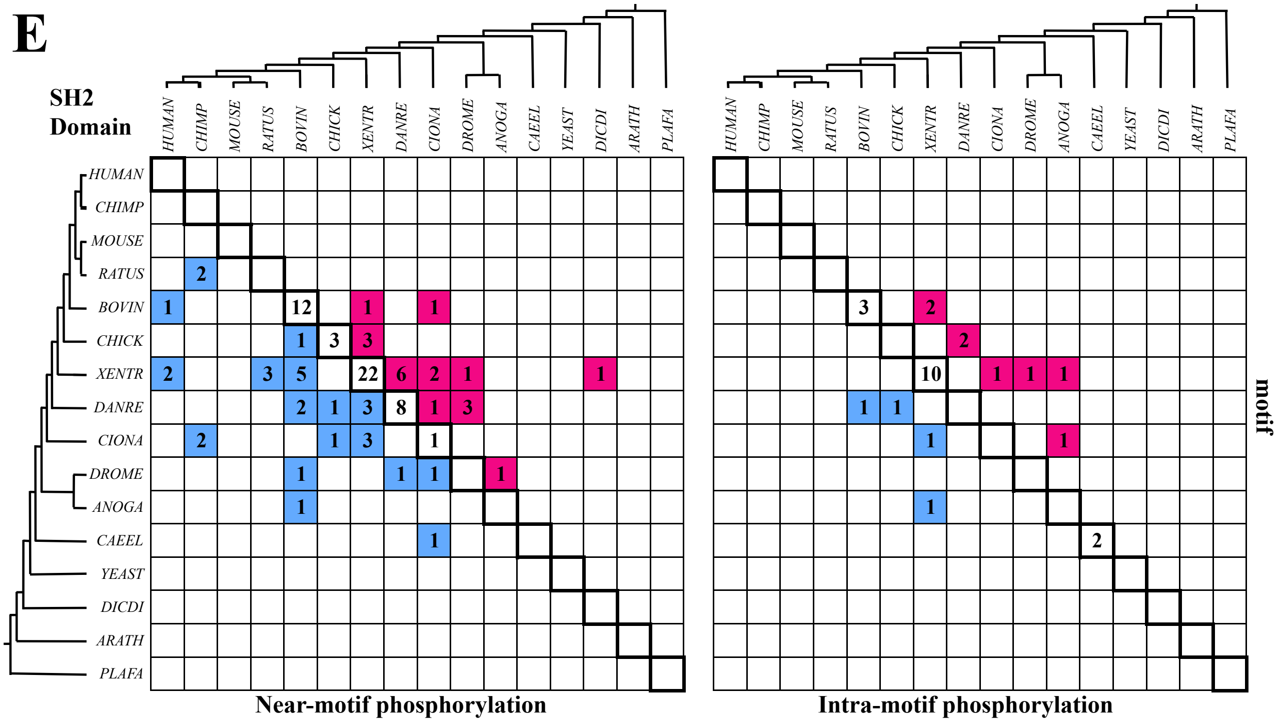
E
SH2 Domain
HUMAN CHIMP MOUSE RATUS BOVIN CHICK XENTR DANRE CIONA DROME ANOGA CAEEL YEAST DICDI ARATH PLAFA
Near-motif phosphorylation
Intra-motif phosphorylation
motif
