# Supplementary material for: A Dynamic View of Domain-Motif Interactions
Source: PLoS Comput Biol. 2012 Jan 12;8(1):e1002341. doi: 10.1371/journal.pcbi.1002341 (PMC3257277; doi:10.1371/journal.pcbi.1002341)
Supplement: Table S1 — Counts and statistical significance of the coupling between domain-motif interactions and phosphorylations. (PDF) [file pcbi.1002341.s003.pdf]

Table S1: Counts and statistical significance of the coupling between domain-motif interactions and phosphorylations

	Phosphorylation			motif-bound domains		Total number of motifs	# motifs that are only intra-phosphorylated	# motifs that are both intra- and near-phosphorylated	# motifs that are only near-phosphorylated	Total number of intra/near phosphorylation	Statistical significance	
	LTP	HTP	LTP+ HTP	LTP	LTP+ HTP						Intra-motif phosphorylation	near-motif phosphorylation
PDZ	■			■		89	7	6	9	22	0.0072	0.0144
		■		■		89	12	7	15	34	0.0192	<0.0012
			■		■	95	10	14	14	38	<0.0012	0.0016
WW	■			■		62	9	18	8	35	<0.0012	0.012
		■		■		62	7	7	5	19	NONE	NONE
			■		■	120	16	45	15	76	<0.0012	NONE
SH3	■			■		70	3	8	22	33	NONE	0.0012
		■		■		70	1	8	21	30	NONE	0.0012
			■		■	378	12	36	73	121	NONE	NONE
SH2	■			■		330	132	139	11	282	<0.0012	0.0012
		■		■		330	76	94	38	208	<0.0012	<0.0012
			■		■	482	137	270	21	428	0.0048	<0.0012

Several combinations of the domain-motif interaction data and the phosphorylation data are shown. The sources of integrated data are indicated by the black boxes on the left side of the table. For instance, the first line represents the integration of low-throughput phosphorylation data with low throughput PDZ domain interaction data. Abbreviations: LTP: low throughput methods; HTP: high throughput methods; LTP+HTP: evidence from high throughput or low throughput resource. The last two columns show the statistical significance of the coupling between motifs and phosphorylation events (see Methods in main text).
