# Supplementary material for: A Dynamic View of Domain-Motif Interactions
Source: PLoS Comput Biol. 2012 Jan 12;8(1):e1002341. doi: 10.1371/journal.pcbi.1002341 (PMC3257277; doi:10.1371/journal.pcbi.1002341)
Supplement: Table S2 — Identification of human protein segments that display a dual motif that can act as a phosphorylation double switch for SH3 and SH2 domains or for SH2 and WW domains. (PDF) [file pcbi.1002341.s004.pdf]

Table S2: Identification of human protein segments that display a dual motif that can act as a phosphorylation double switch for SH3 and SH2 domains or for SH2 and WW domains

		Strict scheme		Less strict scheme		
		Class I WW	SH3-1	SH3-2	SH3-3	SH3-4
		PPx**Y**	PxxD**Y**	[RKY]xxPxxP	PxxPx[KR]	[PV]xxP
SH2 (Grb2)	**Y**xNx	*JUN_HUMAN(167-173)*<br>CASL_HUMAN(109-115)<br>US6NL_HUMAN(629-635)	IRS2_HUMAN(974-981)<br>JAG1_HUMAN(1135-1142)<br>GIT2_HUMAN(588-595)<br>LR37B_HUMAN(898-905)<br>RBM19_HUMAN(165-172)	*WASF3_HUMAN(337-343)*<br>GCM2_HUMAN(359-365)	NONE	*RET_HUMAN(1088-1093)*<br>ROR1_HUMAN(784-789)
SH2 (Ptp2)	**Y**[IV]x[VILP]	*DAG1_HUMAN(889-895)*<br>LAP4A_HUMAN(207-213)<br>MIF_HUMAN(34-40)<br>K1462_HUMAN(337-343)	GEMI2_HUMAN(167-174)	STA5B_HUMAN(668-674)<br>MYPT1_HUMAN(496-502)<br>LIPB2_HUMAN(835-841)<br>SCML2_HUMAN(506-512)<br>SND1_HUMAN(421-427)<br>MUTA_HUMAN(231-237)	NONE	*DAG1_HUMAN(891-895)*<br>RBM34_HUMAN(70-74)<br>PKP1_HUMAN(213-217)<br>RPGR1_HUMAN(928-932)<br>NDC80_HUMAN457-461)<br>K1462(339-343)
SH2 (Src)	**Y**[QDEVAIL][DENPYHI][IPVGAHS]	*EZRI_HUMAN(475-481)*<br>*NTRK1_HUMAN(788-794)*<br>LAP4A_HUMAN(227-233)<br>SCAM3_HUMAN(50-56)<br>DDR1_HUMAN(481-487)<br>CAN7_HUMAN(624-630)	*PTN11_HUMAN(300-307)*<br>*CD3E_HUMAN(184-191)*<br>*HCLS1_HUMAN(374-381)*<br>*HCLS1_HUMAN(393-400)*<br>PTN6_HUMAN(297-304)<br>GRK6_HUMAN(157-164)<br>MATR3(198-205)	*GAB1_HUMAN(307-313)*<br>*B3AT_HUMAN(21-27)*<br>*3BP2_HUMAN(183-189)*<br>MPDZ_HUMAN(717-723)<br>PKP4_HUMAN(478-484)<br>TRIP6_HUMAN(123-129)<br>PARD3_HUMAN(1321-1327)<br>MMP16_HUMAN(52-58)<br>CNO6L_HUMAN(10-16)<br>PHF10_HUMAN(196-202)<br>ABLM3_HUMAN(538-544)<br>CASS4_HUMAN(195-201)	ERG_HUMAN(258-264)	*CD28_HUMAN(208-212)*<br>*EZRI_HUMAN(477-481)*<br>*P63_HUMAN(289-293)*<br>GRWD1_HUMAN(32-36)<br>CING_HUMAN(1177-1181)<br>MTA2_HUMAN(482-486)<br>K1462_HUMAN(339-343)<br>SF3A1_HUMAN(455-459)<br>DSG2_HUMAN(967-971)<br>LAP4A_HUMAN(229-233)<br>RBM34_HUMAN(70-74)<br>WWC3_HUMAN(642-646)<br>BOP1_HUMAN(219-223)<br>RPGR1_HUMAN(928-932)<br>ACHG_HUMAN(511-515)<br>PKP1_HUMAN(213-217)
SH2 (Stat3)	**Y**xxQ	LAP2_HUMAN(974-980)<br>SOX18_HUMAN(5-11)<br>PREX2_HUMAN(1555-1561)	STAP2_HUMAN(318-325)<br>CC03_HUMAN(634-641)	NONE	NONE	ZO1_HUMAN(897-901)<br>CTND1_HUMAN(256-260)

SH2 (Stat5)	**Y**[VLTFIC]	*ERBB4_HUMAN(1053-1057)*<br>*SH23A_HUMAN(228-232)*<br>*EPOR_HUMAN(501-505)*<br>ERBB4_HUMAN(1032-1036)<br>SYNPO_HUMAN(562-566)<br>LAP4B_HUMAN(364-368)<br>FUBP3_HUMAN(540-544)<br>HGS_HUMAN(234-238)<br>INP4B_HUMAN(789-793)<br>NLGN1_HUMAN(775-780)<br>RU2B_HUMAN(148-152)<br>SG223_HUMAN(156-160)	*ICK_HUMAN(155-160)*<br>OCLN_HUMAN(311-316)<br>LYN_HUMAN(488-493)<br>LAP2_HUMAN(1100-1105)<br>MAK_HUMAN(155-160)<br>PTK6_HUMAN(110-115)<br>RAB1B_HUMAN(3-8)<br>US6NL_HUMAN(725-730)<br>CDKAL_HUMAN(144-149)<br>FXRD2_HUMAN(29-34)	*RPB1_HUMAN(1838-1844)*<br>CS021_HUMAN(428-434)<br>KCNC3_HUMAN(574-580)<br>SND1_HUMAN(421-427)<br>MUTA_HUMAN(231-237)<br>PDIP3_HUMAN(236-242)<br>ETV6_HUMAN(17-23)<br>SCML2_HUMAN(506-512)<br>RRMJ1_HUMAN(262-268)<br>MMP16_HUMAN(52-58)<br>STA5B_HUMAN(668-674)<br>LIPB2_HUMAN(835-841)<br>PURA1_HUMAN(384-390)<br>ROBO1_HUMAN(1587-1593)<br>DCP1A_HUMAN(310-316)<br>VIR_HUMAN(217-223)<br>TEX10_HUMAN(623-629)<br>MYPT1_HUMAN(496-502)<br>SFRS9_HUMAN(214-220)<br>ERRFI_HUMAN(395-401)	*IRS4_HUMAN(699-704)*<br>CC14C_HUMAN(238-243)<br>SH3R1_HUMAN(455-460)	*RPB1_HUMAN(1901-1904)*<br>*HCK_HUMAN(50-53)*<br>*DCC_HUMAN(1362-1365)*<br>*INSR_HUMAN(1010-1013)*<br>*GAB2_HUMAN(475-478)*<br>GRWD1_HUMAN(32-35)<br>NUMB_HUMAN(14-17)<br>ARAF_HUMAN(23-26)<br>LAP4A_HUMAN(229-232)<br>RBM34_HUMAN(70-73)<br>WWC3_HUMAN(642-645)<br>ABL2_HUMAN(567-570)<br>K1462_HUMAN(339-342)<br>NDC80_HUMAN(457-460)<br>SFR2B_HUMAN(43-46)<br>RPGR1_HUMAN(928-931)<br>SYQ_HUMAN(476-479)<br>CCDC6_HUMAN(355-358)<br>ACHG_HUMAN(511-514)<br>GSTP1_HUMAN(7-10)<br>PKP1_HUMAN(213-216)
SH2 (Crk)	**Y**xxP	PTN21_HUMAN(566-572)<br>WIPF2_HUMAN(252-258)<br>K1522_HUMAN(675-681)	NCK1_HUMAN(264-271)	*CDN1B_HUMAN(88-94)*<br>*CBL_HUMAN(774-780)*<br>*EMD_HUMAN(19-25)*<br>*RAD51_HUMAN(315-321)*<br>*BCAR1_HUMAN(165-171)*<br>*ASPG_HUMAN(178-184)*<br>*ACK1_HUMAN(860-866)*<br>*3BP2_HUMAN(183-189)*<br>*FGFR1_HUMAN(463-469)*<br>*TAU_HUMAN(514-520)*<br>*DOK1_HUMAN(398-404)*<br>*WASF3_HUMAN(248-254)*<br>*SH22A_HUMAN(280-286)*<br>*DOK1_HUMAN(377-383)*<br>*YAP1_HUMAN(357-363)*<br>*TRIP6_HUMAN(55-61)*<br>*NUAK2_HUMAN(267-273)*<br>*CDN1B_HUMAN(88-94)*<br>MPDZ_HUMAN(717-723)<br>ADA22_HUMAN(880-886)<br>ZO1_HUMAN(1435-1441)<br>WRIP1_HUMAN(111-117)<br>ETV6_HUMAN(17-23)<br>SCML2_HUMAN(506-512)<br>RRMJ1_HUMAN(262-268)	*CD28_HUMAN(208-213)*<br>RB_HUMAN(786-793)<br>ERG_HUMAN(258-263)<br>WIPF2_HUMAN(251-258)	FAK1_HUMAN(718-723)<br>TTDN1_HUMAN(11-16)<br>PKP1_HUMAN(213-217)<br>RPGR1_HUMAN(928-832)<br>K1462_HUMAN(339-343)<br>WWC3_HUMAN(642-646)

			GCM2_HUMAN(359-365)<br>BIG3_HUMAN(1406-1412)<br>FGFR2_HUMAN(466-472)<br>FA21B_HUMAN(351-358)<br>LIPB2_HUMAN(835-841)<br>NRK_HUMAN(985-991)<br>DCP1A_HUMAN(310-316)<br>OTU7B_HUMAN(779-785)<br>PSB4_HUMAN(22-28)<br>BRD1_HUMAN(127-133)<br>PKP4_HUMAN(478-484)<br>SVIL_HUMAN(1317-1323)<br>PARD3_HUMAN(1321-1327)<br>PTN21_HUMAN(564-572)<br>PGAM4_HUMAN(119-125)<br>K1522_HUMAN(678-684)<br>CASS4_HUMAN(244-250)<br>KLF3_HUMAN(106-112)<br>RIF1_HUMAN(404-410)<br>CNO6L_HUMAN(10-16)<br>PK3CD_HUMAN(440-446)<br>PHF10_HUMAN(196-202)<br>LPHN3_HUMAN(170-176)<br>ERRFI_HUMAN(395-401)<br>SHB_HUMAN(297-303)<br>KS6A4_HUMAN(342-348)<br>CS021_HUMAN(428-434)<br>KCNC3_HUMAN(574-580)<br>SND1_HUMAN(421-427)<br>MUTA_HUMAN(231-237)<br>ZFHX4_HUMAN(2662-2668)<br>ATAD2_HUMAN(322-328)<br>TRIP6_HUMAN(123-129)<br>PDIP3_HUMAN(236-242)<br>TM1L2_HUMAN(200-206)<br>DOK4_HUMAN(255-261)<br>US6NL_HUMAN(804-810)<br>MMP16_HUMAN(52-58)<br>STA5B_HUMAN(668-674)<br>ATN1_HUMAN(729-735)<br>SFR19_HUMAN(238-244)<br>ABLM3_HUMAN(538-544)<br>RBM5_HUMAN(712-718)<br>HDAC3_HUMAN(404-410)<br>YTHD1_HUMAN(317-323)<br>TRPS1_HUMAN(1080-1086)		

				KI2S5_HUMAN(126-132)<br>PGAM1_HUMAN(119-125)<br>ROBO1_HUMAN(1587-1593)<br>CASS4_HUMAN(131-137)<br>VIR_HUMAN(217-223)<br>XRN1_HUMAN(1394-1400)<br>TEX10_HUMAN(623-629)<br>MYPT1_HUMAN(496-502)<br>DPYL1_HUMAN(504-510)<br>FAK1_HUMAN(441-447)<br>NDC1_HUMAN(469-475)<br>LSR_HUMAN(615-621)<br>SMS2_HUMAN(24-30)<br>SFRS9_HUMAN(214-220)<br>CASS4_HUMAN(195-201)		
Total(*)		31(7)	26(5)	126(23)	8(2)	53(10)

Each table cell includes a protein segment that may bind SH2 and SH3 (or WW) domains. For example, the red-framed table entry details three protein segments (present in JUN, NEDD9 and USP6NL proteins) that obey the combined sequence pattern PPxYxNx (composed of the $SH2^{Grb2}$ YxNx pattern and the class I WW PPxY pattern – see Figure 3 in the main text for all dual sequence patterns). The third and fourth table columns detail the strict scheme analysis, and the three last columns detail the less-strict analysis results. Protein names (Swiss-Prot accessions or gene names) in italic font mean that the phosphorylation has highly reliable evidence.

(*) Numbers in parenthesis indicate the count of dual sequence pattern hits that are phosphorylated according to our high reliability phosphorylation dataset.
