# Supplementary material for: A Dynamic View of Domain-Motif Interactions
Source: PLoS Comput Biol. 2012 Jan 12;8(1):e1002341. doi: 10.1371/journal.pcbi.1002341 (PMC3257277; doi:10.1371/journal.pcbi.1002341)
Supplement: Table S3 — Proteins that are predicted to be involved in ‘double switches’ based on experimental protein-protein interaction data. (PDF) [file pcbi.1002341.s005.pdf]

Table S3: Proteins that are predicted to be involved in ‘double switches’ based on experimental protein-protein interaction data

SH2 motifs	Class I WW	SH3-1
Grb2	CASL_HUMAN<br>• O75815 (SH2)<br>• P16333 (SH2)<br>• P07948 (SH2)<br>• P46108 (SH2)<br>• P46109 (SH2)<br>• P06239 (SH2)<br>• Q96J02 (WW)<br>JUN_HUMAN<br>• P40763 (SH2)<br>• P42224 (SH2)<br>• Q14765 (SH2)<br>• Q13526 (WW)	IRS2_HUMAN<br>• Q5T4P3 (SH2)<br>• O15524 (SH2)<br>• Q06124 (SH2)<br>• P19174 (SH2, SH3-1)
Ptp2	DAG1_HUMAN<br>• P06241 (SH2)<br>• P62993 (SH2)<br>• P16333 (SH2)<br>• P12931 (SH2)<br>• P27986 (SH2)<br>• P11532 (WW)<br>• P46939 (WW)	
Src	EZRI_HUMAN<br>• P27986 (SH2)<br>• P07332 (SH2)<br>• P43405 (SH2)<br>• P07332 (SH2)<br>• P46940 (WW)	CD3E_HUMAN<br>• P43405 (SH2)<br>• P43403 (SH2)<br>• P27986 (SH2, SH3-2)<br>• P16333 (SH2, SH3-1)<br>• O43639 (SH2, SH3-1)<br>• Q8TE68 (SH3-1)<br>• Q8TE67 (SH3-1)<br>• Q12929 (SH3-1)<br>• Q9H6S3 (SH3-1)<br>HCLS1_HUMAN<br>• Q9H788 (SH2)<br>• P62993 (SH2, SH3-1)<br>• P090769 (SH2, SH3-1)<br>• P07948 (SH2, SH3-1)<br>PTN6_HUMAN<br>• O60674 (SH2)<br>• P43403 (SH2)<br>• P51692 (SH2)<br>• P43405 (SH2)<br>• Q13094 (SH2)<br>• Q8WV28 (SH2)<br>• P27986 (SH2, SH3-2)<br>• P62993 (SH2, SH3-1)<br>• P07948 (SH2, SH3-1)<br>• P15498 (SH2, SH3-1)<br>• P06239 (SH2, SH3-1)<br>PTN11_HUMAN<br>• P40763 (SH2)<br>• O60674 (SH2)<br>• O14543 (SH2)<br>• Q92835 (SH2)<br>• P51692 (SH2)<br>• P42229 (SH2)<br>• Q14451 (SH2)<br>• O00459 (SH2)<br>• P27986 (SH2, SH3-2)<br>• P46109 (SH2, SH3-1, SH3-2)<br>• P62993 (SH2, SH3-1)<br>• P16885 (SH2, SH3-1)<br>• P56945 (SH3-1)

Stat5	SYNPO_HUMAN<br>• P62993 (SH2)<br>• Q96QZ7 (WW)<br>ERBB4_HUMAN<br>• O43639 (SH2)<br>• P42684 (SH2)<br>• P20936 (SH2)<br>• P46108 (SH2)<br>• P46109 (SH2)<br>• P62993 (SH2)<br>• P16333 (SH2)<br>• P00519 (SH2)<br>• Q06124 (SH2)<br>• P29353 (SH2)<br>• P51692 (SH2)<br>• P42229 (SH2)<br>• P43405 (SH2)<br>• O00459 (SH2)<br>• P46937 (WW)<br>• Q96J02 (WW)	OCLN_HUMAN<br>• P07947 (SH2, SH3-1)<br>• A2A3H9 (SH3-2)<br>• Q07157 (SH3-2)<br>LYN_HUMAN<br>• P29350 (SH2)<br>• O60674 (SH2)<br>• Q92835 (SH2)<br>• P29353 (SH2)<br>• P43405 (SH2)<br>• Q13094 (SH2)<br>• P12931 (SH2, SH3-1)<br>• P16885 (SH2, SH3-1)<br>• P07948 (SH2, SH3-1)<br>• P42680 (SH2, SH3-1)<br>• P56945 (SH3-1)<br>• A4D174 (SH3-1)<br>• P14317 (SH3-1)<br>• P78352 (SH3-1)<br>• Q14511 (SH3-1)<br>• Q15642 (SH3-1)<br>• O75563 (SH3-1)<br>• Q86WV1 (SH3-1)
Crk	WIPF2_HUMAN<br>• O43639 (SH2)<br>• Q9NZC7 (WW)	NCK1_HUMAN<br>• O60674 (SH2)<br>• O15524 (SH2)<br>• O14512 (SH2)<br>• P52757 (SH2)<br>• Q13094 (SH2)<br>• Q8WV28 (SH2)<br>• P06241 (SH2, SH3-1)<br>• P46108 (SH2, SH3-1, SH3-2)<br>• P42684 (SH2, SH3-1)<br>• P20936 (SH2, SH3-1)<br>• P00519 (SH2, SH3-1)<br>• O43281 (SH3-1)<br>• Q14511 (SH3-1)<br>• A7KAX9 (SH3-1)<br>• Q9NZQ3 (SH3-1)<br>• Q14185 (SH3-1)<br>• P56945 (SH3-1)<br>• Q9ULH1 (SH3-1)<br>• Q8IZP0 (SH3-1)<br>• O43150 (SH3-1)<br>• Q8IZD9 (SH3-2)<br>• Q9UPZ8 (SH3-2)<br>• Q9UPX8 (SH3-2)<br>• O94885 (SH3-2)<br>• O15117 (SH3-2)<br>• Q9BYB0 (SH3-2)

Table row titles list five different SH2 motifs: $SH2^{Grb2}$, $SH2^{Ptp2}$, $SH2^{Src}$, $SH2^{Stat5}$ and $SH2^{Crk}$. Table columns list Class I WW and SH3 motifs. Each table cell includes potential double switches: The proteins that include the two motifs are shown in capital letters of their Swissprot ID (these proteins originate in Table S2). Each of these motif-containing proteins is documented to interact with human proteins that include WW/SH2/SH3 domains (bullets; shown as Swissprot accessions). Domain content of the latter proteins is brought in parentheses. For instance, the first table entry details CASL_HUMAN as having both $SH2^{Grb2}$ and Class I WW motifs. This protein is also documented as interacting with O75815 (that includes an SH2 domain) and with Q96J02 (that includes a WW domain). Domain assignment was done using Pfam definitions [1].

1. Finn RD, Mistry J, Tate J, Coggill P, Heger A, et al. (2010) The Pfam protein families database. Nucleic Acids Res 38: D211-222.
