# Supplementary material for: A Dynamic View of Domain-Motif Interactions
Source: PLoS Comput Biol. 2012 Jan 12;8(1):e1002341. doi: 10.1371/journal.pcbi.1002341 (PMC3257277; doi:10.1371/journal.pcbi.1002341)
Supplement: Table S4 — Phosphorylation specificity switch for proteins containing domains of the PDZ family. (PDF) [file pcbi.1002341.s006.pdf]

Table S4: Phosphorylation specificity switch for proteins containing domains of the PDZ family

A

		HtrA1 (1/1)	Magi-2 (6/6)
1	NLKAGRETTV(Kif1b)	24634	-1
	CLDTSRETDL(Kcna5)	-1	22260

		SAP97 (3/3)	Scrb1 (2/4)
2	SPLHSLETSL(Atp2b1)	43518	-1
	SNAKAVETDV(Kcna4)	-1	28774

		Gm1582 (2/3)	SAP97 (1/3)
3	PLATCDETSI(Sema4f)	97014	-1
	CLDTSRETDL(Kcna5)	-1	65366

		Gm1582 (2/3)	Semcap3 (1/2)
4	PLATCDETSI(Sema4f)	97014	-1
	PRPLRRESEI(Kcnj2)	-1	45847

		Gm1582 (2/3)	PTP-BL (2/5)
5	PLATCDETSI(Sema4f)	97014	-1
	SNAKAVETDV(Kcna4)	-1	38848

		Lin7c (1/1)	Semcap3 (1/2)
6	PDSNPEESSV(Sema4c)	41233	-1
	PRPLRRESEI(Kcnj2)	-1	45847

		Lin7c (1/1)	PTP-BL (2/5)
7	VDFPPKESSL(Acvr2a)	11852	-1
	KKMPSIESDV(Grin2a)	-1	52174

		HtrA1 (1/1)	g1-syntrophin (1/1)
8	GNDPDRGTSI(Grid2)	26129	-1
	CLDTSRETDL(Kcna5)	-1	21074

		Semcap3 (1/2)	Chapsyn-110 (3/3)
9	VDFPPKESSL(Acvr2a)	18329	-1
	AHFSSLESEV(Grin2c)	-1	14263

		Chapsyn-110 (3/3)	PSD95 (1/3)
10	ELDEFPETSV(Trpv3)	4948	-1
	EKLSSIESDV(Grin2b)	-1	17859

		MUPP1 (13/13)	Magi-2 (6/6)
11	TNSKQGETTV(Fzd1)	46387	-1
	ANLVKEDSDV(Megalin)	-1	15895

		Lin7c (1/1)	Chapsyn-110 (3/3)
12	VDFPPKESSL(Acvr2a)	11852	-1
	PRPLRRESEI(Kcnj2)	-1	5439

		HtrA1 (1/1)	Gm1582 (2/3)
13	NLKAGRETTV(Kif1b)	24634	-1
	EKLSSIESDV(Grin2b)	-1	92597

		Semcap3 (1/2)	Gm1582 (2/3)
14	NLKAGRETTV(Kif1b)	12485	-1
	AHFSSLESEV(Grin2c)	-1	27870

		g1-syntrophin (1/1)	Magi-2 (6/6)
15	SPLHSLETSL(Atp2b1)	78075	-1
	VNIKKIFTDV(Kcna3)	-1	59433

		Gm1582 (2/3)	TIP-1 (1/1)
16	NVVSERISSV(Htr2c)	97451	-1
	PRPLRRESEI(Kcnj2)	-1	49074

		Lin7c (1/1)	Gm1582 (2/3)
17	DTKNYKQTSV(CRIPT)	41217	-1
	EKLSSIESDV(Grin2b)	-1	92597

		Gm1582 (2/3)	PTP-BL (2/5)
18	TNSKQGETTV(Fzd1)	39246	-1
	KKMPSIESDV(Grin2a)	-1	52174

		HtrA1 (1/1)	TIP-1 (1/1)
19	GNDPDRGTSI(Grid2)	26129	-1
	PRPLRRESEI(Kcnj2)	-1	49074

		Gm1582 (2/3)	PTP-BL (2/5)
20	TNSKQGETTV(Fzd1)	39246	-1
	RRISSLESEV(Grin2c)	-1	27872

		PTP-BL (2/5)	TIP-1 (1/1)
21	NVVSERISSV(Htr2c)	26003	-1
	VRPYRRESEI(Kcnj12)	-1	36503

		Lin7c (1/1)	PTP-BL (2/5)
22	NLKAGRETTV(Kif1b)	7258	-1
	KKMPSIESDV(Grin2a)	-1	52174

		HtrA1 (1/1)	PTP-BL (2/5)
23	TNSKQGETTV(Fzd1)	20699	-1
	EKLSSIESDV(Grin2b)	-1	26446

		Lin7c (1/1)	PTP-BL (2/5)
24	VDFPPKESSL(Acvr2a)	11852	-1
	EKLSSIESDV(Grin2b)	-1	26446

		Gm1582 (2/3)	PSD95 (1/3)
25	ELDEFPETSV(Trpv3)	3133	-1
	KKMPSIESDV(Grin2a)	-1	8176

		Magi-3 (5/5)	Magi-2 (6/6)
26	NVVSERISSV(Htr2c)	37603	-1
	PAGKHMVTEV(Kcna7)	-1	79666

		HtrA1 (1/1)	Magi-3 (5/5)
27	NLKAGRETTV(Kif1b)	24634	-1
	ANLVKEDSDV(Megalin)	-1	91467

		Lin7c (1/1)	PTP-BL (2/5)
28	DTKNYKQTSV(CRIPT)	41217	-1
	KKMPSIESDV(Grin2a)	-1	52174

B

		Scrb1 (3/4)	HtrA3 (1/1)
1	GNDPDRGTSI(Grid2)	37966	-1
	NNNLIKVDSV(Mel1a/b)	-1	34912

C

1		Shank1 (1/1)	Lin7c (1/1)
	RDYKQSSSTL (Grm1)	13595	-1
	TNSKQGETTV (Fzd1)	-1	14635

2		Shank1 (1/1)	Lin7c (1/1)
	RDYKQSSSTL (Grm1)	13595	-1
	VDFPPKESSL (Acvr2a)	-1	11852

3		Shank1 (1/1)	Lin7c (1/1)
	RDYKQSSSTL (Grm1)	13595	-1
	NLKAGRETTV (Kif1b)	-1	7258

4		Shank3 (1/1)	Lrrc7 (1/1)
	EVLDSTTSSL (Grm3)	15260	-1
	GARVPKETAL (Mapk12)	-1	41794

5		Shank3 (1/1)	Lin7c (1/1)
	RDYKQSSSTL (Grm1)	8203	-1
	GARVPKETAL (Mapk12)	-1	11878

6		OMP25 (1/1)	Magi-2 (6/6)
	NTANRRTTPV (Stargazin)	7198	-1
	CLDTSRETDL (Kcna5)	-1	22260

7		Shank1 (1/1)	Scrb1 (3/4)
	AHEDYVTTRL (Trpc4)	379	-1
	SNAKAVETDV (Kcna4)	-1	7414

8		Pdzk1 (1/4)	Scrb1 (3/4)
	GQEEQVTTRL (Trpc5)	14911	-1
	TNSKQGETTV (Fzd1)	-1	33133

The table is divided into sub-tables, each including the following data: (1) Pair-wise alignment of peptide sequences, where the S/T/Y and the D/E residues are colored in red. Protein names are given in parentheses. The interaction-relevant C-terminal residues are in black. (2) Names of proteins (Uniprot or gene names) that include the PDZ domains. Numbers in parentheses indicate the domain identifier (*e.g.* 2/4 means the second out of four PDZ domains in the relevant protein). (3) Affinity values for each domain-peptide interaction. Values represent the equilibrium dissociation constant (nM), as measured using fluorescence polarization. A value of (-1) stands for array negatives (*i.e.* no interaction was detected). All values were taken from [1,2]. The table is split into three sections (A, B and C), representing different positions of the Asp/Glu residue in the pseudo-phosphorylated motifs. **(A)**: Asp/Glu residues are positioned one residue upstream to the C-terminal residue. **(B)**: Asp/Glu residues are positioned two residues upstream to the C-terminal residue. **(C)**: Asp/Glu residues are positioned three residues upstream to the C-terminal residue. Note that 11 different proteins that are involved in nine of the 37 putative double switches (Acvr2a, Atp2b1, Chapsyn-110, Grm1, Grm3, Lin7c, SAP97, Scrb1, SEMCAP3, Shank1 and Trpc5) were experimentally verified as associated with the post-synaptic density [3-12]. Note that this table reports 37 PDZ double-switch cases out of the 82 identified: in cases where several PDZ pairs displayed double-switch binding pattern with a specific peptide-pair, we list in the table the one with the highest affinity values for the 'non-phosphorylated or 'pseudo-phosphorylated' peptide pair. Accordingly, we present 28 out of 60 examples for the 'position (-1)' cases, one case of 'position -2' and 8 out of 20 examples of the 'position (-3)' cases. All peptide pairs are sorted according to sequence similarity, taking into account higher weight for the C-terminal positions. When sequence similarities had almost identical scores, we added the affinity values into the sorting criteria (higher affinities were ranked higher).

Table S4 references

1. Chen JR, Chang BH, Allen JE, Stiffler MA, MacBeath G (2008) Predicting PDZ domain-peptide interactions from primary sequences. Nat Biotechnol 26: 1041-1045.
2. Stiffler MA, Chen JR, Grantcharova VP, Lei Y, Fuchs D, et al. (2007) PDZ domain binding selectivity is optimized across the mouse proteome. Science 317: 364-369.
3. Berman HM, Westbrook J, Feng Z, Gilliland G, Bhat TN, et al. (2000) The Protein Data Bank. Nucleic Acids Res 28: 235-242.
4. Cai C, Li H, Rivera C, Keinanen K (2006) Interaction between SAP97 and PSD-95, two Maguk proteins involved in synaptic trafficking of AMPA receptors. J Biol Chem 281: 4267-4273.
5. DeMarco SJ, Strehler EE (2001) Plasma membrane Ca2+-atpase isoforms 2b and 4b interact promiscuously and selectively with members of the membrane-associated guanylate kinase family of PDZ (PSD95/Dlg/ZO-1) domain-containing proteins. J Biol Chem 276: 21594-21600.
6. Goel M, Sinkins W, Keightley A, Kinter M, Schilling WP (2005) Proteomic analysis of TRPC5- and TRPC6-binding partners reveals interaction with the plasmalemmal Na(+)/K(+)-ATPase. Pflugers Arch 451: 87-98.
7. Klemmer P, Smit AB, Li KW (2009) Proteomics analysis of immuno-precipitated synaptic protein complexes. J Proteomics 72: 82-90.
8. Lu Z, Je HS, Young P, Gross J, Lu B, et al. (2007) Regulation of synaptic growth and maturation by a synapse-associated E3 ubiquitin ligase at the neuromuscular junction. J Cell Biol 177: 1077-1089.
9. Sala C, Piech V, Wilson NR, Passafaro M, Liu G, et al. (2001) Regulation of dendritic spine morphology and synaptic function by Shank and Homer. Neuron 31: 115-130.
10. Shoji-Kasai Y, Ageta H, Hasegawa Y, Tsuchida K, Sugino H, et al. (2007) Activin increases the number of synaptic contacts and the length of dendritic spine necks by modulating spinal actin dynamics. J Cell Sci 120: 3830-3837.
11. Sun Y, Aiga M, Yoshida E, Humbert PO, Bamji SX (2009) Scribble interacts with beta-catenin to localize synaptic vesicles to synapses. Mol Biol Cell 20: 3390-3400.
12. Tamaru Y, Nomura S, Mizuno N, Shigemoto R (2001) Distribution of metabotropic glutamate receptor mGluR3 in the mouse CNS: differential location relative to pre- and postsynaptic sites. Neuroscience 106: 481-503.
