# Supplementary material for: A Dynamic View of Domain-Motif Interactions
Source: PLoS Comput Biol. 2012 Jan 12;8(1):e1002341. doi: 10.1371/journal.pcbi.1002341 (PMC3257277; doi:10.1371/journal.pcbi.1002341)
Supplement: Table S5 — Statistical significance for the PDZ double-switch analysis. (PDF) [file pcbi.1002341.s007.pdf]

Table S5: Statistical significance for the PDZ double-switch analysis

**PDZ^A-pep**	**PDZ^A-pep^p**	**PDZ^B-pep**	**PDZ^B-pep^p**	**Count**	
				**pos(-1)**	**pos(-3)**
x>0	x>0	x>0	x>0	21	0
x>0	x>0	x>0	-1	14	0
x>0	-1	x>0	x>0		
x>0	x>0	-1	x>0	51	4
-1	x>0	x>0	x>0		
x>0	x>0	-1	-1	8	0
-1	-1	x>0	x>0		
x>0	-1	x>0	-1	19	0
x>0	-1	-1	x>0	60[(*)]	20[(*)]
-1	x>0	x>0	-1		
x>0	-1	-1	-1	34	9
-1	-1	x>0	-1		
-1	x>0	-1	x>0	27	9
-1	x>0	-1	-1	34	9
-1	-1	-1	x>0		
-1	-1	-1	-1	13	5
			Total	281	56

Values of -1 and >0 refer to non-binding and binding, respectively. The first four values in each row represent a binding pattern: (1) the binding value for PDZ^A with a non-phosphorylated peptide, (2) the binding value for PDZ^A with a 'pseudo-phosphorylated' peptide, (3) the binding value for PDZ^B with a non-phosphorylated peptide, and (4) the binding value for PDZ^B with a 'pseudo-phosphorylated' peptide. Two complementary patterns (*e.g.* -1, >0, >0, -1 and >0, -1, -1, >0) are considered as one pattern. The fifth and sixth columns include the number of cases for which the specific binding pattern was found (for peptide pairs that are non-phosphorylated/'pseudo phosphorylated' in position -1 or -3, respectively).

[(*)] For 'position (-1)' cases, it is expected that at random 28 cases out of 281 (10%) will show the double switch pattern and 253 cases will show all other patterns. Comparison of the actual counts (60 and 221, respectively) to those expected at random is statistically significant (p=0.0003 by Fischer's exact test). Applying the same analysis and test for 'position (-3)' cases also yields statistically significant results (p=0.0031). Since we found only one case of a 'position (-2)' double switch, no relevant statistical analysis could be performed.
