# Supplementary material for: A Dynamic View of Domain-Motif Interactions
Source: PLoS Comput Biol. 2012 Jan 12;8(1):e1002341. doi: 10.1371/journal.pcbi.1002341 (PMC3257277; doi:10.1371/journal.pcbi.1002341)
Supplement: Table S6 — Statistical significance for the evolutionary path analysis. (PDF) [file pcbi.1002341.s008.pdf]

Table S6: Statistical significance for the evolutionary path analysis

Domain	co-appearance	Stepwise appearance	Statistical significance
WW$^{Class\ IV}$(phosphorylation near the motif)			
Expected	12	65	p=2e-7
Observed	37	40	
WW$^{Class\ IV}$(phosphorylation within the motif)			
Expected	15	37	p=0.005
Observed	28	24	
SH2 (phosphorylation near the motif)			
Expected	21	76	p=6.3e-6
Observed	46	51	
SH2 (phosphorylation within the motif)			
Expected	8	19	*insignificant*
Observed	15	12	
PDZ (phosphorylation near the motif)			
Expected	6	31	p=0.0162
Observed	16	21	
PDZ (phosphorylation within the motif)			
Expected	4	16	p=0.0062
Observed	12	8	
WW$^{Class\ I/II/III}$(phosphorylation near the motif)			
Expected	5	21	*insignificant*
Observed	6	20	
WW$^{Class\ I/II/III}$(phosphorylation within the motif)			
Expected	6	13	p=1.73e-5
Observed	15	4	
SH3 (phosphorylation near the motif)			
Expected	14	86	p=1. 29e-5
Observed	38	62	
SH3 (phosphorylation within the motif)			
Expected	3	23	*insignificant*
Observed	7	19	

The expected and observed counts of the two possible evolutionary paths were computed for all domain-motif types, either within or near the motif. The detailed procedure is presented in the Methods section in the main text. Statistical significance was computed using χ^2 test with Yates correction (results were corrected for multiple testing using Bonferroni correction).
