# Supplementary material for: A Dynamic View of Domain-Motif Interactions
Source: PLoS Comput Biol. 2012 Jan 12;8(1):e1002341. doi: 10.1371/journal.pcbi.1002341 (PMC3257277; doi:10.1371/journal.pcbi.1002341)
Supplement: Table S7 — Disorder levels of the various motifs. (PDF) [file pcbi.1002341.s009.pdf]

Table S7: Disorder levels of the various motifs

Domain type	#motifs	Total # of motif residues	Total # of motif residues that are disordered	Percent disordered residues (motifs)	Percent disordered residues (whole proteins)	Percent disordered residues (20 flanking residues)
SH3	70	808	662	81.93%	31.63%	74.79%
PDZ	89	939	662	70.50%	18.61%	37.26%
WW	62	1,703	1044	61.30%	32.66%	65.92%
SH2	330	5,419	2,374	43.8%	21.07%	49.44%

The disorder level of motifs was predicted by the IUPred algorithm [1]. To this end, we considered only motifs that were determined by low-throughput methods, and assumed that each residue that was assigned with disorder value above 0.5 is disordered. Note that the predicted disorder of the whole proteins (sixth column) is lower than the regions flanking the motifs (seventh column), and that the regions flanking the motifs are less disordered than the motifs themselves (fifth column). We used the motif disorder level as a threshold for finding randomly-positioned motifs.

1. Dosztanyi Z, Csizmok V, Tompa P, Simon I: IUPred: web server for the prediction of intrinsically unstructured regions of proteins based on estimated energy content. *Bioinformatics* 2005, 21(16):3433-3434.
